# Further contributions to the Coleoptera fauna of New Brunswick with an addition to the fauna of Nova Scotia, Canada

**DOI:** 10.3897/zookeys.573.7327

**Published:** 2016-03-24

**Authors:** Reginald P. Webster, Vincent L. Webster, Chantelle A. Alderson, Cory C. Hughes, Jon D. Sweeney

**Affiliations:** 1 24 Mill Stream Drive, Charters Settlement, NB, Canada E3C 1X1; 2 Natural Resources Canada, Canadian Forest Service - Atlantic Forestry Centre, 1350 Regent St., P.O. Box 4000, Fredericton, NB, Canada E3B 5P7

**Keywords:** Coleoptera, new records, Canada, New Brunswick, Nova Scotia, Lindgren funnel trap

## Abstract

This paper treats 134 new records of Coleoptera for the province of New Brunswick, Canada from the following 41 families: Gyrinidae, Carabidae, Dytiscidae, Histeridae, Leiodidae, Scarabaeidae, Scirtidae, Buprestidae, Elmidae, Limnichidae, Heteroceridae, Ptilodactylidae, Eucnemidae, Throscidae, Elateridae, Lampyridae, Cantharidae, Dermestidae, Bostrichidae, Ptinidae, Cleridae, Melyridae, Monotomidae, Cryptophagidae, Silvanidae, Laemophloeidae, Nitidulidae, Endomychidae, Coccinellidae, Corylophidae, Latridiidae, Tetratomidae, Melandryidae, Mordellidae, Tenebrionidae, Mycteridae, Pyrochroidae, Aderidae, Scraptiidae, Megalopodidae, and Chrysomelidae. Among these, the following four species are newly recorded from Canada: *Dirrhagofarsus ernae* Otto, Muona & McClarin (Eucnemidae), *Athous equestris* (LeConte) (Elateridae), *Ernobius opicus* Fall (Ptinidae), and *Stelidota coenosa* Erichson (Nitidulidae). The Family Limnichidae is newly reported for New Brunswick, and one species is added to the fauna of Nova Scotia. *Stephostethus productus* Rosenhauer (Latridiidae), Tetratoma (Abstrulia) variegata Casey (Tetratomidae), and *Chauliognathus marginatus* (Fabricius) (Cantharidae) are removed from the faunal list of New Brunswick, and additional records of *Lacconotus punctatus* LeConte (Mycteridae) are presented and discussed. Lindgren funnel traps provided specimens for 104 (78%) of the species and were the sole source of specimens for 89 (66%) of the species reported here, suggesting they are a very useful tool for sampling Coleoptera fauna in the forests of New Brunswick.

## Introduction

In recent years, the Coleoptera of New Brunswick has been studied intensively. In a series of papers published in a Special Issue of ZooKeys (179) on the biodiversity and ecology of the Coleoptera of New Brunswick, Canada, edited by Robert Anderson and Jan Klimaszewski, 320 species of Coleoptera were newly reported from the province in the following 59 families: Gyrinidae, Carabidae, Dytiscidae (Webster and DeMerchant 2012a); Histeridae (Webster et al. 2012c); Geotrupidae, Scarabaeidae (Webster et al. 2012e); Eucinetidae, Scirtidae (Webster et al. 2012f); Buprestidae (Webster and DeMerchant 2012b); Dryopidae, Elmidae, Psephenidae, Ptilodactylidae (Webster and DeMerchant 2012c); Eucnemidae (Webster et al. 2012g); Elateridae (Webster et al. 2012h); Lycidae (Webster et al. 2012i); Dermestidae, Endecatomidae, Bostrichidae, Ptinidae (Webster et al. 2012s); Trogossitidae, Cleridae, Melyridae (Webster et al. 2012j); Silvanidae, Laemophloeidae (Webster et al. 2012k); Sphindidae, Erotylidae, Monotomidae, Cryptophagidae (Webster et al. 2012l); Kateretidae, Nitidulidae, Cerylonidae, Endomychidae, Coccinellidae, Latridiidae (Webster et al. 2012m); Mycetophagidae, Tetratomidae, Melandryidae (Webster et al. 2012n); Mordellidae, Ripiphoridae (Webster et al. 2012o); Tenebrionidae, Zopheridae (Webster et al. 2012q); Stenotrachelidae, Oedemeridae, Meloidae, Mycteridae, Boridae, Pythidae, Pyrochroidae, Anthicidae, Aderidae (Webster et al. 2012p); Cerambycidae (Webster et al. 2012r); Megalopodidae, Chrysomelidae (Webster et al. 2012b); Anthribidae, Brentidae, Dryophthoridae, Brachyceridae, Curculionidae (Webster et al. 2012a). In these papers, new habitat and biological data were presented for many of the species, as well as an updated list of the species from each family known to occur in New Brunswick. Recently, Pelletier and Hébert (2014) reviewed the Cantharidae of eastern Canada and reported 19 species new to New Brunswick. Most recently, Webster et al. (2016) newly reported an additional 16 species of Cerambycidae for the province. These baseline biodiversity data are important for documentation of changes in our ecosystems due to human intervention and climate change.

During the last several years, additional new provincial records have been accumulated from the families Gyrinidae, Carabidae, Dytiscidae, Histeridae, Leiodidae, Scarabaeidae, Scirtidae, Buprestidae, Elmidae, Limnichidae, Heteroceridae, Ptilodactylidae, Eucnemidae, Throscidae, Elateridae, Lampyridae, Cantharidae, Dermestidae, Bostrichidae, Ptinidae, Cleridae, Melyridae, Monotomidae, Cryptophagidae, Silvanidae, Laemophloeidae, Nitidulidae, Endomychidae, Coccinellidae, Corylophidae, Latridiidae, Tetratomidae, Melandryidae, Mordellidae, Tenebrionidae, Mycteridae, Pyrochroidae, Aderidae, Scraptiidae, Megalopodidae, and Chrysomelidae. The purpose of this paper is to report these new records.

## Methods and conventions

**Collection methods.** Various methods were employed to collect the specimens reported in this study. Details are outlined in Webster et al. (2009, Appendix) and Webster et al. (2012d). Many specimens were from Lindgren funnel trap samples from a study to improve methods for survey and detection of exotic and potentially invasive bark and wood-boring beetles (Cerambycidae and Curculionidae). These traps are visually similar to tree trunks and are often effective for sampling species of Coleoptera that live in microhabitats associated with standing trees (Lindgren 1983). Between 2009 and 2015, Lindgren funnel traps were deployed at 27 sites (24–64 traps per site). At many sites, starting in 2012, traps were deployed in the upper canopy as well as in the understory, usually in equal numbers, although at a few sites only canopy traps or understory traps were used. Canopy traps were 10–20 m above the ground, whereas understory traps were 1–1.5 m above the ground (i.e., 30–50 cm from the bottom of the collecting cup to the ground). In both cases, traps were suspended from rope such that the trap was at least 1 m from the main stem of trees and at least 30 m from another trap. Traps were baited with various combinations of lures for detecting Cerambycidae. However, data on attractants were not collected for non-target species. See Webster et al. (2012r), Hughes et al. (2014), and Webster et al. (2016) for additional details of the lures and methods used to deploy Lindgren traps and collect samples. A description of the habitat was recorded for all specimens collected during this survey. Locality and habitat data are presented as on labels for each record. Two labels were used on many specimens: one that included the locality, collection date, and collector, and one with macro- and microhabitat data and collection method. Information from the two labels is separated by a double slash (//) in the data presented from each specimen.

**Specimen preparation and determination.** Males of some species were dissected to confirm their identities. The genital structures were dehydrated in absolute alcohol and either mounted in Canada balsam on celluloid microslides or glued onto cards that were then pinned with the specimen from which they originated. Most specimens reported in this study were determined by the senior author using various keys to the families or genera treated in this publication. Some specimens in the families Ptinidae, Cryptophagidae, and Melyridae were compared to specimens from the Canadian National Collection (CNC) to confirm their names. A number of species in the families Scarabaeidae, Elateridae, Buprestidae, Dermestidae, Ptinidae, Elateridae, Melyridae, Mordellidae, Tenebrionidae, and Chrysomelidae were determined by curators at the CNC.

**Distribution.** Every species is cited with current distribution in Canada and Alaska, using abbreviations for the state, provinces, and territories. New records for New Brunswick are indicated in **bold** under **Distribution in Canada and Alaska**. The following abbreviations are used in the text:

AK Alaska


MB Manitoba


YT Yukon Territory


ON Ontario


NT Northwest Territories


QC Quebec


NU Nunavut


NB New Brunswick


BC British Columbia


PE Prince Edward Island


AB Alberta


NS Nova Scotia


SK Saskatchewan


NF & LB Newfoundland and Labrador*


*Newfoundland and Labrador are each treated separately under the current Distribution in Canada and Alaska.

USA state abbreviations follow those of the US Postal Service. Acronyms of collections examined or where specimens reside referred to in this study are as follows:

AFC
 Atlantic Forestry Centre, Fredericton, New Brunswick, Canada 


CNC
 Canadian National Collection of Insects, Arachnids and Nematodes, Ottawa, Ontario, Canada 


KNPC
 Kouchibouguac National Park Collection, New Brunswick, Canada 


NBM
 New Brunswick Museum, Saint John, New Brunswick, Canada 


RWC
 Reginald P. Webster Collection, Charters Settlement, New Brunswick, Canada 


## Results and discussion

In this account, we newly report 133 species and one new subspecies from NB. Four of these species are new for Canada. Additional records are given for the rarely collected *Lacconotus punctatus* LeConte (Mycteridae). One species is newly recorded from NS. Eighty-nine of the new records were collected exclusively from Lindgren funnel traps as part of a study to improve methods for survey and detection of exotic and potentially invasive bark and wood-boring beetles (Cerambycidae, Curculionidae). Another 15 species were detected using both Lindgren funnel traps and other sampling methods. The above data indicate that Lindgren traps are a very useful tool for sampling Coleoptera fauna in the forests of NB. Thirty-two species were found using methods such as litter sampling, light trapping, or use of an aquatic dip net. Below, we present the details of the new records.

### Species accounts

All records below are species newly recorded for NB or NS, Canada, unless noted otherwise (additional records). Species indicated by a † are adventive to Canada; species with a * are Holarctic; species with a ‡ are either adventive or Holarctic. The determination that a species was a new record is based on information in the print version of Bousquet et al. (2013). The family-level classification used below follows Bouchard et al. (2011).

### Suborder Adephaga

#### Family Gyrinidae Latreille, 1810

The Gyrinidae of the Maritime Provinces were reviewed by Majka and Kenner (2009). They reported 17 species for NB, including four new provincial records. Webster and DeMerchant (2012a) added two more species. Here, we add another two species to the faunal list of the province.

##### Subfamily Gyrininae Latreille, 1810

###### Tribe Enhydrusini Régimbart, 1882

####### Gyrinus (Gyrinus) marginellus

Taxon classificationAnimaliaColeopteraGyrinidae

Fall, 1922

######## Material examined.

**New Brunswick, Sunbury Co.**, Juvenile Settlement at S. Branch Oromocto River, 45.5341°N, 66.6096°W, 27.VI.2006, M.-A. Giguère & R. Webster // Gravel bottomed river in trailing vegetation (1 ♂ [dissected], RWC). **York Co.**, Douglas, near Nashwaaksis River, 45.9842°N, 66.6908°W, 1.VII.2003, R.P. Webster // Mixed forest, small sandy bottomed stream (2 ♂♂ [dissected], RWC).

######## Distribution in Canada and Alaska.

QC, **NB** (Bousquet et al. 2013).

####### Gyrinus (Gyrinus) ventralis

Taxon classificationAnimaliaColeopteraGyrinidae

Kirby, 1837

######## Material examined.

**New Brunswick, Sunbury Co.**, Maugerville, Portobello Creek N.W.A., 45.8992°N, 66.4248°W, 27.V.2004, R.P. Webster // Silver maple forest, margin of slow [flowing] river (2 ♂♂ [dissected], RWC); Burton, near Sunpoke Lake, 45.7657°N, 66.5563°W, 17.VII.2007, R.P. Webster // Lake margin (1 ♂ [dissected], RWC).

######## Distribution in Canada and Alaska.

SK, ON, QC, **NB** (Bousquet et al. 2013).

#### Family Carabidae Latreille, 1810

The Carabidae of NB were reviewed by Webster and Bousquet (2008), and they reported 50 species new to the province. Later, Webster and DeMerchant (2012a) added another four species. Most recently, *Carabus auratus auratus* Linnaeus was newly recorded for Canada and NB by Lewis et al. (2015). Here, we add three additional species to the faunal list of the province.

##### Subfamily Harpalinae Bonelli, 1810

###### Tribe Lebiini Bonelli, 1810

####### Subtribe Lebiina Bonelli, 1810

######## Lebia (Lebia) solea

Taxon classificationAnimaliaColeopteraCarabidae

Hentz, 1830

######### Material examined.

**New Brunswick, York Co.**, Keswick Ridge, 45.9962°N, 66.8781°W, 22.V-4.VI.2014, C. Alderson & V. Webster // Mixed forest, Lindgren funnel trap in canopy (1, RWC).

######### Distribution in Canada and Alaska.

SK, MB, ON, QC, **NB**, NS (Bousquet et al. 2013).

###### Tribe Oodini Laferté-Sénectère, 1851

####### Oodes fluvialis

Taxon classificationAnimaliaColeopteraCarabidae

LeConte, 1863

######## Material examined.

**New Brunswick, Queens Co.**, Scotchtown, Grand Lake Meadows P.N.A., 45.8762°N, 66.1816°W, 16.VI.2013, 17.VI.2013, R.P. Webster // Lake margin, sifting flood debris (1, NBM; 3, RWC); same locality data, collection date, and collector but sweeping foliage (1, RWC).

######## Distribution in Canada and Alaska.

ON, QC, **NB** (Bousquet et al. 2013).

###### Tribe Platynini Bonelli, 1810

####### Platynus (Batenus) cincticollis

Taxon classificationAnimaliaColeopteraCarabidae

(Say, 1823)

######## Material examined.

**New Brunswick, Queens Co.**, Grand Lake Meadows P.N.A. 45.8227°N, 66.1209°W, 12.IV-3.VI.2011, M. Roy & V. Webster, coll. // Old silver maple forest and seasonally flooded marsh, Lindgren funnel trap (1 ♂ [dissected], RWC).

######## Distribution in Canada and Alaska.

ON, QC, **NB**, NS (Bousquet et al. 2013).

#### Family Dytiscidae Leach, 1815

Webster (2008) reviewed the Dytiscidae of NB, adding 18 species to the faunal list. Another species was added by Webster and DeMerchant (2012a). Below, three more dytiscid species are newly reported for the province.

##### Subfamily Copelatinae Branden, 1885

###### Copelatus glyphicus

Taxon classificationAnimaliaColeopteraDytiscidae

(Say, 1823)

####### Material examined.

**New Brunswick, Gloucester Co.**, Bathurst, Daly Point Nature Preserve, 47.6392°N, 65.6098°W, 28.V-15.VI.2015, C. Alderson & V. Webster // Mixed forest, green Lindgren funnel trap 1 m high (1, RWC). **Kent Co.**, Kouchibouguac National Park, 46.8087°N, 64.9078°W, 21-27.V.2015, C. Alderson & V. Webster // Poplar/red maple stand, Lindgren funnel trap, 1 m high (1, RWC). **York Co.**, Canterbury, Eel River P.N.A., 45.8967°N, 67.6343°W, 8-21.V.2014, C. Alderson & V. Webster // Old-growth eastern white cedar swamp & fen, Lindgren funnel trap (1, RWC); Fredericton, Odell Park, 45.9584°N, 66.6802°W, 12-22.V.2014, C. Alderson & V. Webster // Old mixed forest, Lindgren funnel trap 1 m high under trees (2, RWC).

####### Distribution in Canada and Alaska.

ON, QC, **NB**, NS, PE, NF (Bousquet et al. 2013).

####### Comments.

Larson et al. (2000) mentioned that *Copelatus glyphicus* (Say) was abundant during early August in gravel pit pools and tire ruts in peaty soils in NS and NB. However, this species was not listed as occurring in NB in the checklist of the species occurring in Canada (Table 1, p. 15) and there are no dots on the distribution map for this species in the province (Map 2, p. 52) in Larson et al. (2000). The records above confirm the presence of this species in NB. All specimens from NB were captured in Lindgren funnel traps.

##### Subfamily Agabinae C.G. Thomson, 1867

###### Agabus (Acatodes) bicolor

Taxon classificationAnimaliaColeopteraDytiscidae

(Kirby, 1837)

####### Material examined.

**New Brunswick, Restigouche Co.**, Sport Camp Brook Rd., 47.9582°N, 68.0183°W, 30.VII.2012, R.P. Webster, & M. Turgeon // Logging road through spruce & cedar forest, under log in dried puddle on roadside (1 ♂ [dissected], RWC).

####### Distribution in Canada and Alaska.

AK, YT, NT, BC, AB, AK, MB, ON, **NB**, NS (Bousquet et al. 2013).

###### Ilybius ignarus

Taxon classificationAnimaliaColeopteraDytiscidae

(LeConte, 1862)

####### Material examined.

**New Brunswick**, Canterbury, Eel River P.N.A., 45.8966°N, 67.6345°W, 8-21.V.2014, C. Alderson & V. Webster // Old-growth eastern white cedar swamp & fen, Lindgren funnel trap (1, RWC).

####### Distribution in Canada and Alaska.

ON, QC, **NB**, NS (Bousquet et al. 2013).

### Suborder Polyphaga

#### Superfamily Hydrophiloidea Latreille, 1802

##### Family Histeridae Gyllenhal, 1808

Webster et al. (2012c) added 18 species of Histeridae to the faunal list of NB in their review of this family for the province. Here, we add another two species. All specimens were captured in Lindgren funnel traps.

###### Subfamily Abraeinae MacLeay, 1819

####### Tribe Teretriini Bickhardt, 1914

######## Teretrius latebricola

Taxon classificationAnimaliaColeopteraHisteridae

Lewis, 1901

######### Material examined.

**New Brunswick, Carleton Co.**, Jackson Falls, “Bell Forest”, 46.2200°N, 67.7231°W, 8-23.V.2012, C. Alderson & V. Webster // Rich Appalachian hardwood forest, Lindgren funnel traps in canopy of *Tilia americana* (1, RWC). **Queens Co.**, C.F.B. Gagetown, 45.7516°N, 66.1866°W, 23.V-4.VI.2013, 4-17.VI.2013, C. Alderson & V. Webster // Old mixed forest with *Quercus rubra*, Lindgren funnel traps in canopy of *Quercus rubra* (1, AFC; 1, RWC). **York Co.**, Douglas, Currie Mountain, 45.9844°N, 66.7592°W, 27.V-10.VI.2013, C. Alderson & V. Webster // Mixed forest with *Quercus rubra*, Lindgren funnel trap in canopy of *Quercus rubra* (1, AFC); Keswick Ridge, 45.9962°N, 66.8781°W, 4-19.VI.2014, C. Alderson & V. Webster // Mixed forest, Lindgren funnel trap in canopy (1, RWC).

######### Distribution in Canada and Alaska.

ON, QC, **NB** (Bousquet et al. 2013).

######### Comments.

All specimens of *Teretrius latebricola* Lewis from NB were captured in Lindgren funnel traps in the canopy of various tree species. This species occurs under bark of hardwoods and pines, often in galleries of xylophagus Coleoptera such as Bostrichidae, Ptinidae, and Eucnemidae (Bousquet and Laplante 2006).

###### Subfamily Tribalinae Bickhardt, 1914

####### Epierus pulicarius

Taxon classificationAnimaliaColeopteraHisteridae

Erichson, 1834

######## Material examined.

**New Brunswick, Queens Co.**, Cranberry Lake P.N.A., 46.1125°N, 65.6075°W, 7-13.VII.2011, M. Roy & V. Webster // Old red oak forest, Lindgren funnel trap (1, RWC); **York Co.**, Keswick Ridge, 45.9962°N, 66.8781°W, 30.VI-16.VII.2015, C. Alderson & V. Webster // Mixed forest, Lindgren funnel trap 1 m high under trees (1, RWC).

######## Distribution in Canada and Alaska.

ON, QC, **NB** (Bousquet et al. 2013).

#### Superfamily Staphylinoidea Latreille, 1802

##### Family Leiodidae Fleming, 1821

The Leiodidae of Atlantic Canada and NB were reviewed by Majka and Langor (2008). Eight species were newly recorded for NB in this review. Here, we add another species to the provincial list.

###### Subfamily Leiodinae Fleming, 1821

####### Tribe Agathidiini Westwood, 1838

######## Agathidium depressum

Taxon classificationAnimaliaColeopteraLeiodidae

Fall, 1934

######### Material examined.

**New Brunswick, Carleton Co.**, Jackson Falls, “Bell Forest”, 46.2200°N, 67.7231°W, 13-25.IV.2012, R. Webster, J. Sweeney, & C. Hughes // Rich Appalachian hardwood forest, Lindgren funnel trap in canopy of *Acer saccharum* (1 ♂ [dissected], RWC). **York Co.**, Fredericton, Odell Park, 45.9571°N, 66.6650°W, 1-15.VI.2012, C. Alderson & V. Webster // Old-growth eastern hemlock forest, 1 m high under *Betula alleghaniensis* (1 ♂ [dissected], RWC); Canterbury, Eel River P.N.A., 45.8967°N, 67.6343°W, 8-21.V.2014, C. Alderson & V. Webster // Old-growth eastern white cedar swamp & fen, Lindgren funnel trap (1 ♂ [dissected], RWC).

######### Distribution in Canada and Alaska.

AK, BC, AB, SK, MB, QC, **NB**, NS (Bousquet et al. 2013).

######### Comments.

This species has been collected from the slime molds, *Stemonitis fusca* Roth, and *Badhamia* sp. (Myxomycetes) in Alaska and from various kinds of litter, rotten logs, and pine duff (Wheeler and Miller 2005). The NB specimens were captured in Lindgren funnel traps.

#### Superfamily Scarabaeoidea Latreille, 1802

##### Family Scarabaeidae Latreille, 1802

Webster et al. (2012e) added 12 species of Scarabaeidae to the faunal list of NB in their review of the Geotrupidae and Scarabaeidae of the province. Here, we add another five species of Scarabaeidae to the provincial list.

###### Subfamily Aphodiinae Leach, 1815

####### Tribe Aphodiini Leach, 1815

######## Agoliinus guttatus

Taxon classificationAnimaliaColeopteraScarabaeidae

(Eschschultz, 1823)

######### Material examined.

**New Brunswick, York Co.**, Charters Settlement, 45.8439°N, 66.7275°W, 5.V.2006, R.P. Webster // Mixed forest, entrance to porcupine den, in porcupine dung (10, RWC).

######### Distribution in Canada and Alaska.

AK, YT, BC, AB, SK, MB, ON, QC, **NB**, NS, NF (Bousquet et al. 2013).

######## Chilothorax distinctus

Taxon classificationAnimaliaColeopteraScarabaeidae

(O. F. Müller, 1776)†

######### Material examined.

**New Brunswick, York Co.**, Douglas, Keswick River at Rt. 105, 45.9922°N, 66.8326°W, 9.V.2006, R.P. Webster // Upper river margin, in deer dung on sand/clay soil (1, RWC).

######### Distribution in Canada and Alaska.

BC, AB, SK, MB, ON, QC, **NB**, NS (Bousquet et al. 2013).

######## Dialytes ulkei

Taxon classificationAnimaliaColeopteraScarabaeidae

Horn, 1875

######### Material examined.

**New Brunswick, York Co.**, Douglas, Currie Mountain, 45.9844°N, 66.7592°W, 7-19.VIII.2013, C. Alderson & V. Webster // Mixed forest with *Quercus rubra*, Lindgren funnel trap 1 m high under *Quercus rubra* (1, RWC); same locality and collectors but 45.9832°N, 66.7564°W, 7-19.VIII.2013 // Old *Pinus strobus* stand, Lindgren funnel trap 1 m high under *Pinus strobus* (1, RWC); Keswick Ridge, 45.9962°N, 66.8781°W, 3-18.VII.2014, 13-28.VIII.2014, 28.VIII-11.IX.2014, C. Alderson & V. Webster // Mixed forest, Lindgren funnel traps 1 m high under trees (1, AFC: 3, RWC); Fredericton, Odell Park, 45.9584°N, 66.6802°W, 17.VII-1.VIII.2014, C. Alderson & V. Webster // Old mixed forest, Lindgren funnel trap 1 m high under trees (1, AFC).

######### Distribution in Canada and Alaska.

ON, QC, **NB** (Bousquet et al. 2013).

######### Comments.

All specimens from NB were captured in Lindgren funnel traps. *Dialytes ulkei* Horn is often found in deer (Cervidae: *Odocoileus*) dung (Gordon and Skelley (2007).

######## Planolinus tenellus

Taxon classificationAnimaliaColeopteraScarabaeidae

(Say, 1823)

######### Material examined.

**New Brunswick, York Co.**, Charters Settlement, 45.8267°N, 66.7343°W, 4.X.2005, R.P. Webster (1, RWC).

######### Distribution in Canada and Alaska.

AK, YT, NT, BC, AB, SK, MB, ON, QC, **NB** (Bousquet et al. 2013).

###### Subfamily Melolonthinae Leach, 1819

####### Tribe Melolonthini Leach, 1819

######## Subtribe Rhizotrogina Burmeister, 1855

######### Amphimallon majale

Taxon classificationAnimaliaColeopteraScarabaeidae

(Razoumowsky, 1789)†

########## Material examined.

**New Brunswick, York Co.**, Fredericton, Odell Park, 45.9539°N, 66.6666°W, 24.VI-9.VII.2013, 9-24.VII.2013, 7-19.VIII.2013, C. Alderson & V. Webster // Hardwood forest, Lindgren funnel traps in canopy of *Populus grandifolia* (1), in canopy of *Fagus grandifolia* (1), 1 m high under trees (1) (3, RWC); Douglas, N.B. Walking Trail, 45.9819°N, 66.7568°W, 1-16.VII.2015, C. Alderson & V. Webster // Hardwood forest, Lindgren funnel trap 1 m high under trees (1, RWC).

########## Distribution in Canada and Alaska.

BC, ON, QC, **NB** (Bousquet et al. 2013).

########## Comment.

All specimens of this adventive species were caught in Lindgren funnel traps. This species could become a potential lawn pest in NB.

#### Superfamily Scirtoidea Fleming, 1821

##### Family Scirtidae Fleming, 1821

The Eucinetidae and Scirtidae of NB were reviewed by Webster at al. (2012f). They added five species of Scirtidae to the faunal list of the province, including *Sarabandus robustus* (LeConte), which was newly recorded for Canada. Here, we add *Sacodes thoracica* (Guérin-Méneville) to the provincial list.

###### Subfamily Scirtinae Fleming, 1821

####### Sacodes thoracica

Taxon classificationAnimaliaColeopteraScirtidae

(Guérin-Méneville, 1843)

######## Material examined.

**New Brunswick, York Co.**, Fredericton, Odell Park, 45.9539°N, 66.6666°W, 24.VI-9.VII.2013, C. Alderson & V. Webster // Hardwood forest, Lindgren funnel trap in canopy of *Populus grandifolia* (1, RWC); same locality, collectors, and forest type but 45.9508°N, 66.6723°W, 29.VI-14.VII.2015 // Lindgren funnel trap in canopy (1, RWC); Keswick Ridge, 45.9962°N, 66.8781°W, 18-30.VI.2015, C. Alderson & V. Webster // Hardwood forest, green Lindgren funnel trap in canopy of trees (1, AFC).

######## Distribution in Canada and Alaska.

ON, QC, **NB** (Bousquet et al. 2013).

######## Comment.

All three specimens of this species were captured in Lindgren funnel traps in the canopy of trees.

#### Superfamily Buprestoidea Leach, 1815

##### Family Buprestidae Leach, 1815

Nine species of Buprestidae were added to the faunal list of NB by Webster and DeMerchant (2012b) in their review of this family for the province. Recently, Lewis (2015) newly reported *Buprestis consularis* Gory. We add another seven species to the faunal list in this publication. Most of the new records were detected using Lindgren funnel traps.

###### Subfamily Chrysochroinae Laporte, 1835

####### Tribe Dicercini Gistel, 1848

######## Subtribe Dicercina Gistel, 1848

######### Dicerca callosa callosa

Taxon classificationAnimaliaColeopteraBuprestidae

Casey, 1909

########## Material examined.

**New Brunswick, Kent Co.**, Kouchibouguac N.P., near Callander Beach, 46.8066°N, 64.9064°W, 18.VII.2014, R.P. Webster // Jack pine forest, on trunk of *Populus tremuloides* (1, RWC).

########## Distribution in Canada and Alaska.

AK, YT, NT, BC, AB, SK, MB, ON, QC, **NB** (Bousquet et al. 2013).

####### Tribe Poecilonotini Jakobson, 1913

######## Subtribe Poecilonotina Jakobson, 1913

######### Poecilonota ferrea

Taxon classificationAnimaliaColeopteraBuprestidae

(Melsheimer, 1845)

########## Material examined.

**New Brunswick, Queens Co.**, C.F.B. Gagetown, 45.7516°N, 66.1866°W, 12-28.VIII.2013, C. Alderson & V. Webster // Old mixed forest with *Quercus rubra*, Lindgren funnel trap in canopy of *Populus grandifolia* (1, RWC). **Restigouche Co.**, Jacquet River Gorge P.N.A., 47.8257°N, 66.0764°W, 19.VIII-2.IX.2014, C. Alderson & V. Webster // Old *Populus balsamifera* stand near river, Lindgren funnel trap in canopy of *Populus balsamifera* (1, NBM). **Sunbury Co.**, Gilbert Island, 45.8770°N, 66.2954°W, 20.VI-5.VII.2013, 5-17.VII.2013, C. Alderson, C. Hughes, & V. Webster // Hardwood forest, Lindgren funnel traps in canopy of *Populus tremuloides* (6, AFC; 8, RWC). **York Co.**, Keswick Ridge, 45.9962°N, 66.8781°W, 3-18.VI.2015, 18-30.VI.2015, C. Alderson & V. Webster // Mixed forest, Lindgren funnel traps in canopy (2, AFC).

########## Distribution in Canada and Alaska.

BC, AB, SK, MB, ON, QC, **NB** (Bousquet et al. 2013).

########## Comments.

All specimens of *Poecilonota ferrea* from NB were captured in Lindgren funnel traps in the canopy of *Populus tremuloides* Michx. (quaking aspen), *Populus balsamifera* L. (balsam poplar) or *Populus grandifolia* Michx. (largetooth aspen). Paiero et al. (2012) list *Populus tremuloides* and *Populus trichocarpa* Torr. & Gray (black cottonwood) as larval hosts of this infrequently collected species.

###### Subfamily Buprestinae Leach, 1815

####### Tribe Anthaxiini Gory & Laporte, 1839

######## Anthaxia (Haplanthaxia) viridifrons

Taxon classificationAnimaliaColeopteraBuprestidae

Gory, 1841

######### Material examined.

**New Brunswick, Sunbury Co.**, Gilbert Island, 45.8770°N, 66.2954°W, 20.VI-5.VII.2013, C. Alderson, C. Hughes, & V. Webster // Hardwood forest, Lindgren funnel traps in canopy of *Ulmus americana* (2, AFC; 3, RWC).

######### Distribution in Canada and Alaska.

MB, ON, QC, **NB** (Paiero et al. 2012).

######### Comments.

*Anthaxia viridifrons* was not listed as occurring in NB or Canada by Bousquet et al. (2013). *Anthaxia viridifrons* and *Anthaxia viridicornis* (Say) were treated as distinct species by MacRae (2006), who noted that some Canadian records of *Anthaxia virdicornis* might pertain to *Anthaxia viridifrons*. Paiero et al. (2012) followed this treatment and reported *Anthaxia viridifrons* from MB, ON, and QC.

###### Subfamily Agrilinae Laporte, 1835

####### Tribe Agrilini Laporte, 1835

######## Agrilus juglandis

Taxon classificationAnimaliaColeopteraBuprestidae

Knull, 1920

######### Material examined.

**New Brunswick, Sunbury Co.**, Gilbert Island, 45.8770°N, 66.2954°W, 12-29.VI.2012, 11-25.VII.2012, C. Alderson, C. Hughes, & V. Webster // Hardwood forest, Lindgren funnel traps in canopy of *Juglans cinerea* (19) and 1 m high under *Juglans cinerea* (2) (9, AFC; 1, CNC; 3, NBM; 8, RWC [8 ♂ dissected]). **York Co.**, Keswick Ridge, 45.9962°N, 66.8781°W, 30.VI-16.VII.2015, C. Alderson & V. Webster // Mixed forest, Lindgren funnel trap in canopy (1 ♂ [dissected], CNC).

######### Distribution in Canada and Alaska.

ON, QC, **NB** (Bousquet et al. 2013).

######### Comments.

Most specimens (19 out of 22) of *Agrilus juglandis* Knull were captured in Lindgren funnel traps in the canopy of butternut, *Juglans cinerea* (L.), the larval host of this species (Paiero et al. 2012). This species is apparently mostly active in the canopy of its host.

######## Agrilus masculinus

Taxon classificationAnimaliaColeopteraBuprestidae

Horn, 1891

######### Material examined.

**New Brunswick, Carleton Co.**, Jackson Falls, “Bell Forest”, 46.2200°N, 67.7231°W, 7-21.VI.2012, C. Alderson & V. Webster // Rich Appalachian hardwood forest, Lindgren funnel traps in canopy of *Juglans cinerea* (1 ♂ [dissected], RWC). **Queens Co.**, C.F.B. Gagetown, 45.7516°N, 66.1866°W, 19.VI-2.VII.2015, C. Alderson & V. Webster // Old mixed forest with *Quercus rubra*, Lindgren funnel trap 1 m high under trees (1, AFC). **Restigouche Co.**, Jacquet River Gorge P.N.A., 47.8257°N, 66.0764°W, 22.VII-5.VIII.2014, C. Alderson & V. Webster // Old *Populus balsamifera* stand near river, Lindgren funnel trap in canopy of *Populus balsamifera* (1 ♂ [dissected], RWC). **York Co.**, Fredericton, Odell Park, 45.9571°N, 66.6650°W, 10-26.VII.2012, C. Alderson & V. Webster // Old-growth eastern hemlock forest, in canopy of *Betula alleghaniensis* (1, RWC); same locality and collectors but 45.9539°N, 66.6666°W, 10-24.VI.2013, 24.VI-9.VII.2014, 9-24.VII.2013, 7-19.VIII.2013 // Hardwood stand, Lindgren funnel traps in canopy (3 [2 ♂ dissected], AFC; 5 [2 ♂ dissected], RWC); Keswick Ridge, 45.9962°N, 66.8781°W, 19.VI-3.VII.2014, 30.VI-16.VII.2015, 16-29.VII.2015, 29.VII-3.VIII.2015, 13-27.VIII.2015, C. Alderson & V. Webster // Mixed forest, Lindgren funnel traps in canopy (4), green Lindgren funnel traps 1 m high (7) (11 [2 ♂ dissected], AFC; (1 ♂ [dissected], RWC).

######### Distribution in Canada and Alaska.

SK, MB, QC, **NB** (Bousquet et al. 2013).

######### Comments.

All specimens of *Agrilus masculinus* from NB were captured in Lindgren funnel traps.

######## Agrilus osburni

Taxon classificationAnimaliaColeopteraBuprestidae

Knull, 1937

######### Material examined.

**New Brunswick**, **York Co.**, Fredericton, Odell Park, 45.9539°N, 66.6666°W, 24.VI-9.VII.2014, C. Alderson & V. Webster // Hardwood stand, Lindgren funnel trap in canopy (1 ♂ [dissected], RWC).

######### Distribution in Canada and Alaska.

ON, QC, **NB** (Bousquet et al. 2013).

######## Agrilus pseudocoryli

Taxon classificationAnimaliaColeopteraBuprestidae

Fisher, 1928

######### Material examined.

**New Brunswick, York Co.**, Canterbury, near “Browns Mtn Fen”, 45.8978°N, 67.6273°W, 3.VII.2005, M-A. Giguère & R.P. Webster // Mixed forest, on foliage of *Corylus cornuta* (3 [1 ♂ dissected], RWC).

######### Distribution in Canada and Alaska.

MB, ON, QC, **NB** (Bousquet et al. 2013).

######### Comments.

This species has been treated by some authors as a subspecies of *Agrilus politus* (Say), which uses various willow (*Salix*) species as larval hosts (Paiero et al. 2012). Larvae of *Agrilus pseudocoryli* Fischer, in contrast, have been recorded from American hazelnut (*Corylus americana* Walter) and beaked hazelnut (*Corylus cornuta* Marsh.) (Paiero et al. 2012). Specimens from NB were collected on the foliage of beaked hazelnut. We have found specimens of *Agrilus politus* in NB on the foliage of willow, and they have a slightly differently shaped aedeagus from those of *Agrilus pseudocoryli*.

#### Superfamily Byrrhoidea Latreille, 1804

##### Family Elmidae Curtis, 1830

One species of Elmidae, *Promorensia elegans* (LeConte), was added to the NB faunal list by Webster and DeMerchant (2012c) in their review of the NB members of the family. Here, we add *Dubiraphia minima* Hilsenhoff and *Dubiraphia vittata* (Melsheimer).

###### Subfamily Elminae Curtis, 1830

####### Tribe Elmini Curtis, 1830

######## Dubiraphia minima

Taxon classificationAnimaliaColeopteraElmidae

Hilsenhoff, 1973

######### Material examined.

**New Brunswick, Queens Co.**, Grand Lake at Indian Point [Grand Lake Meadow P.N.A.], 45.8713°N, 66.1722°W, 28.VII.2005, R. Capozi & R.P. Webster // Lakeshore/beach, sweeping foliage near lake margin (10, RWC).

######### Distribution in Canada and Alaska.

MB, ON, QC, **NB** (Bousquet et al. 2013).

######## Dubiraphia vittata

Taxon classificationAnimaliaColeopteraElmidae

(Melsheimer, 1844)

######### Material examined.

**New Brunswick, Queens Co.**, Jemseg, 45.8412°N, 66.1195°W, 8-21.VIII.2012, C. Alderson, C. Hughes, & V. Webster // Hardwood woodland near seasonally flooded marsh, Lindgren funnel trap 1 m high under *Quercus macrocarpa* (1, RWC). **Sunbury Co.**, Gilbert Island, 45.8770°N, 66.2954°W, 25.VII-8.VIII.2012, 8-21.VIII.2012, C. Alderson, C. Hughes, & V. Webster // Hardwood forest, Lindgren funnel traps 1 m high under *Juglans cinerea* (10) and 1 m high under *Tilia americana* (1) (2, AFC; 1, NBM; 9, RWC).

######### Distribution in Canada and Alaska.

AB, SK, MB, ON, QC, **NB**, NS (Bousquet et al. 2013).

######### Comments.

All specimens were caught in Lindgren funnel traps in the understory of trees near a large river (Saint John River).

##### Family Limnichidae Erichson, 1846

Members of this small family are riparian and live on streamside plants, emergent vegetation and wood, or in drift material on stream margins and are thought to be herbivores (Shepard 2002). Three species of Limnichidae are known to occur in Canada (Bousquet et al. 2013). Here, we report *Limnichites punctatus* (LeConte) and this family for the first time for NB.

###### Subfamily Limnichinae Erichson, 1846

####### Tribe Limnichini Erichson, 1846

######## Limnichites punctatus

Taxon classificationAnimaliaColeopteraLimnichidae

(LeConte, 1854)

######### Material examined.

**New Brunswick, Carleton Co.**, “Bell Forest Nature Preserve”, 46.2150°N, 67.7190°W, 20.VI.2005, M.-A. Giguère & R.P. Webster // River margin, seepage area, on bare clay (1, RWC); same locality data, 24.VI.2005, J. Edsall & R. Webster // River margin, on firm moist clay near seepage area (7, RWC). **York Co.**, trail to “Browns Mtn. Fen”, 45.8964°N, 67.6273°W, 8.IX.2007, R.P. Webster // Mixed forest (near brook), sweeping roadside foliage (2, RWC).

######### Distribution in Canada and Alaska.

BC, MB, ON, QC, **NB** (Bousquet et al. 2013).

######### Comments.

Most adults of *Limnichites punctatus* were found along a river margin on moist bare clay near a seepage area. Specimens were collected after splashing the clay bank. The splashing caused them to move, making them easier to see on the dark substrate.

##### Family Heteroceridae MacLeay, 1825

The Heteroceridae or variegated mud-loving beetles, as their name implies, are often associated with mud and clay in riparian habitats, including salt marshes (Katovich 2002). Adults live and feed on algae and other organic material in shallow, often horizontal burrows in mud or moist organic sand (Katovich 2002). Katovich (2002) provided more details on the ecology and classification of this family. Bousquet et al. (2013) recorded 28 species from Canada, including five species for NB. Here, we report *Heterocerus subtilis* for the first time for the province.

###### Subfamily Heterocerinae MacLeay, 1825

####### Tribe Heterocerini MacLeay, 1825

######## Heterocerus subtilis

Taxon classificationAnimaliaColeopteraHeteroceridae

W.V. Miller, 1988

######### Material examined.

**New Brunswick, Sunbury Co.**, Maugerville, Portobello Creek N.W.A., 45.8992°N, 66.4248°W, 24.VI.2004, R.P. Webster // Silver maple forest, margin of slow (flowing) river, under litter on muddy soil (1, RWC). **York Co.**, Mazerolle Settlement, 45.8729°N, 66.8311°W, 28.IV.2006, R.P. Webster // Margin of stream (sun-exposed), on mud with sparse vegetation (3, RWC); same locality but 45.8765°N, 66.8260°W, 8.VI.2008, R.P. Webster // Beaver meadow, treading mud on brook margin (6, RWC).

######### Distribution in Canada and Alaska.

MB, ON, QC, **NB** (Bousquet et al. 2013).

##### Family Ptilodactylidae Laporte, 1836

*Anchytarsus bicolor* (Melsheimer) was reported for the first time from NB by Webster and DeMerchant (2012c), which was the first record of the family Ptilodactylidae for the province. Here, we add another member of the family to the provincial list.

###### Subfamily Ptilodactylinae Laporte, 1836

####### Ptilodactyla carinata

Taxon classificationAnimaliaColeopteraPtilodactylidae

Johnson & Freytag, 1978

######## Material examined.

**New Brunswick, York Co.**, Canterbury, Eel River P.N.A., 45.8967°N, 67.6343°W, 2-15.VII.2014, C. Alderson & V. Webster // Old-growth eastern white cedar swamp & fen, Lindgren funnel trap (1 ♂ [dissected], RWC).

######## Distribution in Canada and Alaska.

QC, **NB** (Bousquet et al. 2013)

#### Superfamily Elateroidea Leach, 1815

##### Family Eucnemidae Eschscholtz, 1829

Webster et al. (2012g) reviewed the Eucnemidae of NB, reporting nine new provincial records. Most of the species and specimens reported in that publication were captured in Lindgren funnel traps. Here, we add three more species, including a new Canadian record, *Dirrhagofarsus ernae* Otto, Muona & Mcclarin. All specimens of these three species were captured in Lindgren funnel traps.

###### Subfamily Melasinae Fleming, 1821

####### Tribe Dirhagini Reitter, 1911

######## Sarpedon scabrosus

Taxon classificationAnimaliaColeopteraEucnemidae

Bonvouloir, 1875

######### Material examined.

**New Brunswick, York Co.**, Fredericton, Odell Park, 45.9539°N, 66.6666°W, 27.VII-7.VIII.2013, C. Alderson & V. Webster // Hardwood forest, Lindgren funnel trap 1 m high under trees (1, RWC); Douglas, N.B. Walking Trail, 45.9819°N, 66.7568°W, 29.VII-13.VIII.2015, C. Alderson & V. Webster // Hardwood forest, Lindgren funnel trap 1 m high under trees (1, RWC); Keswick Ridge, 45.9962°N, 66.8781°W, 29.VII-13.VIII.2015, C. Alderson & V. Webster // Mixed forest, Lindgren funnel trap 1 m high under trees (1, RWC).

######### Distribution in Canada and Alaska.

BC, ON, QC, **NB** (Bousquet et al. 2013)

####### Tribe Epiphanini Muona, 1993

######## Dirrhagofarsus ernae

Taxon classificationAnimaliaColeopteraEucnemidae

Otto, Muona & Mcclarin, 2014†

######### Material examined.

**Canada, New Brunswick, Gloucester Co.**, Bathurst, Daly Point Nature Preserve, 47.6392°N, 65.6098°W, 23.VII-5.VIII.2015, C. Alderson & V. Webster // Mixed forest, green Lindgren funnel trap 1 m high (1, RWC). **Sunbury Co.**, Gilbert Island, 45.8770°N, 66.2954°W, 5-17.VII.2013, C. Alderson, C. Hughes, & V. Webster // Hardwood forest, Lindgren funnel traps in canopy of *Populus tremuloides* and *Juglans cinerea* (2, RWC). **York Co.**, Fredericton, Odell Park, 45.9539°N, 66.6666°W, 24.VI-9.VII.2013, 9-24.VII.2013, 24.VII-7.VIII.2013, 7-19.VIII.2013, C. Alderson & V. Webster // Hardwood forest, Lindgren funnel traps in canopy of trees (2, AFC; 8, RWC); same locality and collectors but 45.9484°N, 66.6802°W, 17.VI-3.VII.2014 // Old mixed forest, Lindgren trap in front of tree hole and Lindgren trap 1 m high under trees (1, CNC; 1, AFC); Keswick Ridge, 45.9962°N, 66.8781°W, 29.VII-13.VIII.2015, C. Alderson & V. Webster // Hardwood forest, purple Lindgren funnel trap in canopy (1, AFC).

######### Distribution in Canada and Alaska.

**NB (New Canadian record).**

######### Comments.

*Dirrhagofarsus ernae* was recently described from OH, in the USA and occurs from NH, west to WI, south to MO, AL and VA (Otto et al. (2014). According to Otto et al. (2014), the sudden appearance of this species in the USA suggests that it may be an introduction to North America, possibly a previously unknown species from Asia. In NB, no specimens were encountered prior to 2013 despite intensive sampling with Lindgren funnel traps at many sites, including Odell Park, where it was first encountered during 2013. It is possible that *Dirrhagofarsus ernae* is a recent arrival to NB.

###### Subfamily Macraulacinae Fleutiaux, 1923

####### Tribe Macraulacini Fleutiaux, 1923

######## Isarthrus calceatus

Taxon classificationAnimaliaColeopteraEucnemidae

(Say, 1839)

######### Material examined.

**New Brunswick, York Co.**, Fredericton, Odell Park, 45.9484°N, 66.6802°W, 17.VII-1.VIII.2014, C. Alderson & V. Webster // Old mixed forest, Lindgren funnel trap in front of tree hole (1, RWC).

######### Distribution in Canada and Alaska.

ON, QC, **NB** (Bousquet et al. 2013)

##### Family Throscidae Laporte, 1840

Only eight species of this small family of beetles have been documented from Canada, including two from NB (Bousquet et al. 2013). Majka (2011) reviewed the Throscidae of Atlantic Canada based on examination of specimens in collection from the region. He provided distribution maps, color habitus photographs, and a key to the species occurring in the region. Little is known about the biology of members of this family. Adults are often captured in light traps, passive traps, or netted in late afternoon flights, found in litter samples or collected from foliage, and may be generalist pollen and mold feeders (Yensen 1975, Johnson 2002). Other details on biology, structure, and classification are included in Johnson (2002). Here, we newly record *Trixagus chevrolati* (Bonvouloir) for the province.

###### Trixagus chevrolati

Taxon classificationAnimaliaColeopteraThroscidae

(Bonvouloir, 1859)

####### Material examined.

**New Brunswick, Queens Co.**, Grand Lake Meadows P.N.A. 45.8227°N, 66.1209°W, 19.VII-5.VIII.2011, M. Roy & V. Webster // Old silver maple forest and seasonally flooded marsh, Lindgren funnel trap (1, RWC); same locality data, forest type and trap type but C. Hughes & R. Webster (1, RWC). **Restigouche Co.**, Jacquet River Gorge P.N.A., 47.8257°N, 66.0764°W, 29.V-10.VI.2014, 10-25.VI.2014, 9-22.VII.2014, C. Alderson & V. Webster // Old *Populus balsamifera* stand near river, Lindgren funnel traps under trees (2, AFC; 1, NBM). **Sunbury Co.**, Burton, near Sunpoke Lake, 45.7658°N, 66.5546°W, 29.VII.2007, R.P. Webster // Red oak & red maple forest, m.v. light (1, RWC). **York Co.**, Charters Settlement, 45.8395°N, 66.7391°W, 7.IX.2007, 20.VI.2012, 14.VII.2012, R.P. Webster // Mixed forest, u.v. light (4, RWC).

####### Distribution in Canada and Alaska.

BC, ON, QC, **NB**, NS (Bousquet et al. 2013)

##### Family Elateridae Leach, 1815

The Elateridae occurring in NB were recently reviewed by Webster et al. (2012h). They newly recorded 22 species, removed *Negastrius exiguus* (Randall), and reinstated *Agriotes pubescens* Melsheimer to the provincial list. Here, we add another eight species of Elateridae to the faunal list, including one species that is new to Canada. Lindgren funnel traps captured the specimens for all but one species.

###### Subfamily Agrypninae Candèze, 1857

####### Tribe Agrypnini Candèze, 1857

######## Lacon maculatus

Taxon classificationAnimaliaColeopteraElateridae

(LeConte, 1866)

######### Material examined.

**New Brunswick, Carleton Co.**, Jackson Falls, “Bell Forest”, 46.2200°N, 67.7231°W, 8-23.V.2012, 21.VI-3.VII.2012, C. Alderson & V. Webster // Rich Appalachian hardwood forest, Lindgren funnel traps in canopy of *Tilia americana* (2, RWC). **Queens Co.**, C.F.B. Gagetown, 45.7516°N, 66.1866°W, 4-17.VI.2013, 17.VI-3.VII.2013, 3-15.VII.2013, C. Alderson & V. Webster // Old mixed forest with *Quercus rubra*, Lindgren funnel trap in canopy of *Quercus rubra* (3, AFC; 1, NBM; 4, RWC). **Sunbury Co.**, Gilbert Island, 45.8770°N, 66.2954°W, 12-29.VI.2012, 29.VI-11.VII.2012, 20.VI-5.VII.2013, 5-17.VII.2013, C. Alderson, C. Hughes, & V. Webster // Hardwood forest, Lindgren funnel traps in canopy of *Acer saccharinum* (1), *Fraxinus pennsylvanica* (2), *Juglans cinerea* (2), *Populus tremuloides* (1), *Tilia americana* (1) (5, AFC; 1, RWC; 1, NBM); Sunpoke Lake, 45.7656°N, 66.5550°W, 18.VI-9.VII.2012, C. Alderson & V. Webster // Red oak forest near seasonally flooded marsh, Lindgren funnel traps 1 m high under *Quercus rubra* (2, RWC). **York Co.**, Fredericton, Odell Park, 45.9539°N, 66.6666°W, 24.VI-9.VII.2013, 9-24.VII.2013, C. Alderson & V. Webster // Hardwood stand, Lindgren funnel traps in canopy (1, AFC; 1, NBM).

######### Distribution in Canada and Alaska.

ON, QC, **NB** (Bousquet et al. 2013).

######### Comment.

Most specimens (19 out of 21) of *Lacon maculatus* were captured in Lindgren funnel traps in the canopy of various tree species in hardwood and mixed forests.

###### Subfamily Dendrometrinae Gistel, 1848

####### Tribe Dendrometrini Gistel, 1848

######## Subtribe Dendrometrina Gistel, 1848

######### Athous equestris

Taxon classificationAnimaliaColeopteraElateridae

(LeConte, 1853)

Fig. 1

########## Material examined.

**Canada, New Brunswick, Sunbury Co.**, Gilbert Island, 45.8770°N, 66.2954°W, 20.VI-5.VII.2013, C. Alderson, C. Hughes, & V. Webster // Hardwood forest, Lindgren funnel trap in canopy of *Acer saccharinum* (1, RWC).

########## Distribution in Canada and Alaska.

**NB.** (**New Canadian record)**.

########## Comments.

*Athous equestris* occurs from NJ, west to SD and KS, south to GA and MS in the USA and was considered rare by Becker (1974). Its presence in NB is a surprise, as the closest known locality is in NJ.

**Figure 1.**
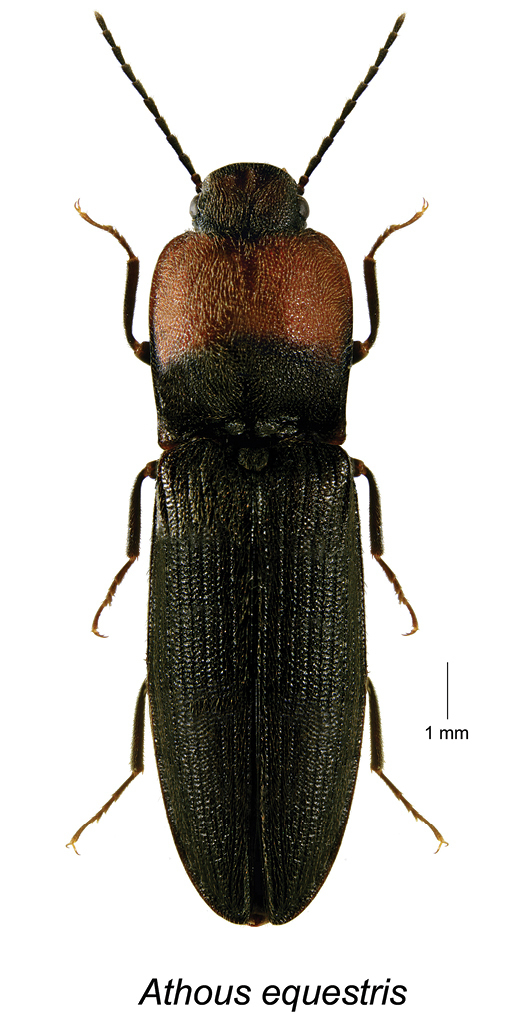
*Athous equestris* (LeConte).

######### Limonius aurifer

Taxon classificationAnimaliaColeopteraElateridae

LeConte, 1853

########## Material examined.

**New Brunswick, York Co.**, Douglas, Currie Mountain, 45.9832°N, 66.7564°W, 7-19.VIII.2013, C. Alderson & V. Webster // Old *Pinus strobus* stand, Lindgren funnel trap in canopy of *Pinus strobus* (1, AFC); Douglas, N.B. Walking Trail, 45.9819°N, 66.7568°W, 20.IV-5.V.2015, C. Alderson & V. Webster // Hardwood forest, Lindgren funnel trap 1 m high under trees (1, RWC).

########## Distribution in Canada and Alaska.

MB, ON, QC, **NB** (Bousquet et al. 2013).

######### Limonius stigma

Taxon classificationAnimaliaColeopteraElateridae

(Herbst, 1806)

########## Material examined.

**New Brunswick, Queens Co.**, C.F.B. Gagetown, 45.7516°N, 66.1866°W, 20.V-4.VI.2015, C. Alderson & V. Webster // Old mixed forest with *Quercus rubra*, Lindgren funnel trap 1 m high under trees (1, RWC). **York Co.**, Douglas, Currie Mountain, 45.9832°N, 66.7564°W, 3-15.V.2013, C. Alderson & V. Webster // Old *Pinus strobus* stand, Lindgren funnel trap 1 m high under *Pinus strobus* (1, RWC); Fredericton, Odell Park, 45.9539°N, 66.6666°W, 15-27.V.2013, 27.V-10.VI.2013, C. Alderson & V. Webster // Hardwood forest, Lindgren funnel traps 1 m high under trees (1, AFC; 2, RWC).

########## Distribution in Canada and Alaska.

ON, QC, **NB** (Bousquet et al. 2013).

######## Subtribe Hemicrepidiina Champion, 1896

######### Harminius triundulatus

Taxon classificationAnimaliaColeopteraElateridae

(Mannerheim, 1853)

########## Material examined.

**New Brunswick, Northumberland Co.**, Upper Graham Plains, 47.1001°N, 66.8154°W, 9-24.VII.2014, C. Alderson & V. Webster // Old black spruce forest, Lindgren funnel trap (1, RWC).

########## Distribution in Canada and Alaska.

AK, YT, BC, AB, SK, ON, QC, **NB** (Bousquet et al. 2013).

####### Tribe Hypnoidini Schwarz, 1906

######## Hypnoidus rivularius

Taxon classificationAnimaliaColeopteraElateridae

(Gyllenhal, 1827)*

######### Material examined.

**New Brunswick, Restigouche Co.**, Wild Goose Lake, 420 m elev., 47.8540°N, 68.3219°W, 20.VI.2011, R.P. Webster & M. Turgeon // Lake margin in leaf & grass litter under alders (2, RWC).

######### Distribution in Canada and Alaska.

AK, YT, NT, BC, AB, SK, MB, QC, **NB**, LB, NF (Bousquet et al. 2013).

####### Tribe Prosternini Gistel, 1856

######## Eanus (Paranomus) decoratus

Taxon classificationAnimaliaColeopteraElateridae

(Mannerheim, 1853)

######### Material examined.

**New Brunswick, Northumberland Co.**, Upper Graham Plains, 47.1001°N, 66.8154°W, 24.VI-9.VII.2014, 9-24.VII.2014, C. Alderson & V. Webster // Old black spruce forest, Lindgren funnel traps (3, AFC; 1, NBM; 10, RWC).

######### Distribution in Canada and Alaska.

AK, YT, NT, BC, AB, SK, MB, ON, QC, **NB**, LB, NF (Bousquet et al. 2013).

###### Subfamily Elaterinae Leach, 1815

####### Tribe Ampedini Gistel, 1848

######## Ampedus linteus

Taxon classificationAnimaliaColeopteraElateridae

(Say, 1839)

######### Material examined.

**New Brunswick, Queens Co.**, Jemseg, 45.8412°N, 66.1195°W, 2-14.V.2012, 14-28.V.2012, 28.V-12.VI.2012, C. Hughes, & R.P. Webster // Hardwood woodland near seasonally flooded marsh, Lindgren funnel traps 1 m high under *Quercus macrocarpa* (5), in canopy of *Quercus macrocarpa* (1) and 1 m high under *Quercus rubra* (7) (5, AFC; 1, CNC; 2, NBM; 5, RWC); same locality data and forest type, 28.V-1.VI.2012, C. Alderson, C. Hughes & V. Webster // Lindgren funnel traps in canopy of *Quercus rubra* (2, RWC). **Sunbury Co.**, Gilbert Island, 45.8770°N, 66.2954°W, 18-28.V.2012, 28.V-12.VI.2012, C. Alderson, C. Hughes, & V. Webster // Hardwood forest, Lindgren funnel traps in canopy of *Tilia americana* (2, RWC); Sunpoke Lake, 45.7656°N, 66.5550°W, 24.V-4.VI.2012, C. Alderson & V. Webster // Red oak forest near seasonally flooded marsh, Lindgren funnel trap in canopy of *Quercus rubra* (1, RWC).

######### Distribution in Canada and Alaska.

ON, QC, **NB** (Bousquet et al. 2013).

##### Family Lampyridae Rafinesque, 1815

Lloyd (2002) provided an overview of the taxonomy, classification, and biology of the Lampyridae of North America. Later, Luk et al. (2011) provided a key to the species of Lampyridae of ON that is applicable to all species occurring in eastern Canada. The Lampyridae of Atlantic Canada were subsequently reviewed by Majka (2012), adding three new species for NB. Using the key by Luk et al. (2011), we identified specimens of *Photinus aquilonius* Lloyd and *Photinus ignitus* Fall from material originally determined as *Photinus ardens* LeConte. Both species are new to NB.

###### Subfamily Lampyrinae Rafinesque, 1815

####### Tribe Lucidotini Lacordaire, 1857

######## Subtribe Photinina LeConte, 1881

######### Photinus aquilonius

Taxon classificationAnimaliaColeopteraLampyridae

Lloyd, 1969

########## Material examined.

**New Brunswick, Sunbury Co.**, Sunpoke Lake, 45.7656°N, 66.5550°W, 18.VI-9.VII.2012, C. Alderson & V. Webster // Red oak forest near seasonally flooded marsh, Lindgren funnel trap 1 m high under *Quercus rubra* (1, RWC); Gilbert Island, 45.8770°N, 66.2954°W, 11-25.VII.2012, C. Alderson, C. Hughes, & V. Webster // Hardwood forest, Lindgren funnel trap 1 m high under *Tilia americana* (1, RWC).

########## Distribution in Canada and Alaska.

MB, ON, QC, **NB**, NS (Bousquet et al. 2013).

######### Photinus ignitus

Taxon classificationAnimaliaColeopteraLampyridae

Fall, 1927

########## Material examined.

**New Brunswick, York Co.** Charters Settlement, 45.8395°N, 66.7391°W, 9.VII.2007, 23.VII.2007, R.P. Webster // Mixed forest, m.v. light (4, RWC); Keswick Ridge, 45.9962°N, 66.8781°W, 18-30.VII.2014, C. Alderson & V. Webster // Field/meadow, Lindgren funnel trap 1 m high (1, RWC).

########## Distribution in Canada and Alaska.

ON, QC, **NB** (Bousquet et al. 2013).

##### Family Cantharidae Imhoff, 1856

Pelletier and Hébert (2014) reviewed the taxonomy, known biology, and distribution the Cantharidae of eastern Canada and the northeastern USA. Members of this family, known as soldier beetles, are common and often occur on foliage and flowers. Most feed on small insects, nectar, and pollen, and some are natural control agents for aphids (Pelletier and Hébert 2014 and references therein). They newly reported 19 species for NB in this publication. Here, we add another four species of Cantharidae to the faunal list of NB and one for NS.

###### Subfamily Cantharinae Imhoff, 1856

####### Tribe Cantharini Imhoff, 1856

######## Cantharis livida

Taxon classificationAnimaliaColeopteraCantharidae

Linnaeus, 1758†

######### Material examined.

**Nova Scotia, Halifax Co.**, Magazine Hill, 44°, 42’, 19.1”N, 63°, 37”, 19.89”W, 30.VI.2014, Sweeney Lab, coll. // High-Low Experiment, Ketols Lure, High Trap (1, AFC).

######### Distribution in Canada and Alaska.

ON, QC, NB, **NS** (Pelletier and Hébert 2014).

######### Comments.

Pelletier and Hébert (2014) reported *Cantharis livida* for the first time for Canada based on records from NB, QC, and ON. This species occurs south to MA and NY in the USA and was introduced from Europe (Pelletier and Hébert 2014). The specimen from Halifax represents the first record for NS.

######## Rhagonycha dichroa

Taxon classificationAnimaliaColeopteraCantharidae

(LeConte, 1851)

######### Material examined.

**New Brunswick, Queens Co.**, Bayard near Nerepis River, 45.4474°N, 66.3326°W, 4.VII.2012, R.P. Webster // River margin, sweeping vegetation on sand bar (2, RWC); same locality but 45.4475°N, 66.3326°W, 4.VII.2014, R.P. Webster // Sweeping marsh/old field near river (1, RWC).

######### Distribution in Canada and Alaska.

ON, **NB** (Pelletier and Hébert 2014).

######## Rhagonycha sylvatica

Taxon classificationAnimaliaColeopteraCantharidae

(Green, 1941)

######### Material examined.

**New Brunswick, Sunbury Co.**, Gilbert Island, 45.8770°N, 66.2954°W, 12.VI.2012, R.P. Webster // Hardwood forest on island in river, sweeping vegetation (1, RWC).

######### Distribution in Canada and Alaska.

ON, QC, **NB** (Bousquet et al. 2013, Pelletier and Hébert 2014).

######## Rhagonycha tantilla

Taxon classificationAnimaliaColeopteraCantharidae

(LeConte, 1881)

######### Material examined.

**New Brunswick, Queens Co.**, C.F.B. Gagetown, 45.7516°N, 66.1866°W, 17.VI-3.VII.2013, C. Alderson & V. Webster // Old mixed forest with *Quercus rubra*, Lindgren funnel traps in canopy of *Quercus rubra* (4, RWC). **Sunbury Co.**, Gilbert Island, 45.8770°N, 66.2954°W, 20.VI-5.VII.2012, C. Alderson, C. Hughes, & V. Webster // Hardwood forest, Lindgren funnel trap in canopy of *Ulmus americana* (1, RWC).

######### Distribution in Canada and Alaska.

AB, SK, MB, ON, QC, **NB** (Bousquet et al. 2013, Pelletier and Hébert 2014).

**Comments.** All specimens of *Rhagonycha tantilla* from the province were caught in Lindgren funnel traps in the canopy of trees.

####### Tribe Podabrini Gistel, 1856

######## Podabrus tricostatus

Taxon classificationAnimaliaColeopteraCantharidae

(Say, 1835)

######### Material examined.

**New Brunswick, York Co.**, Canterbury, Eel River P.N.A., 45.8967°N, 67.6343°W, 20.VI-2.VII.2014, C. Alderson & V. Webster // Old-growth eastern white cedar swamp & fen, Lindgren funnel trap (1, RWC); Keswick Ridge, 45.9962°N, 66.8781°W, 30.VI-16.VII.2015, C. Alderson & V. Webster // Mixed forest, Purple Lindgren funnel trap 1 m high under trees (1, RWC).

######### Distribution in Canada and Alaska.

ON, QC, **NB** (Bousquet et al. 2013, Pelletier and Hébert 2014).

###### Subfamily Chauliognathinae LeConte, 1861

####### Tribe Chauliognathini LeConte, 1861

######## *Chauliognathus marginatus* (Fabricius, 1775) and *Chauliognathus pensylvanicus* (DeGeer, 1774)

*Chauliognathus marginatus* (Fabricius) was reported from NB by Bousquet et al. (2013). This species is known only from extreme southern ON in Canada (Pelletier and Hébert 2014). We were not able to find any records of this species from NB. Interestingly, *Chauliognathus pensylvanicus* (Fabricius), a common and widespread species in NB, was not included for the province by Bousquet et al. (2013). We assume that *Chauliognathus marginatus* was included for NB instead of *Chauliognathus pensylvanicus* (DeGeer) in error. In view of this, *Chauliognathus marginatus* is removed from the faunal list of NB. Pelletier and Hébert (2014) provide supporting data for the occurrence of *Chauliognathus pensylvanicus* in NB.

#### Superfamily Bostrichoidea Latreille, 1802

Webster et al. (2012s) reviewed the Dermestidae, Endecatomidae, Bostrichidae, and Ptinidae fauna of NB and newly reported two species of Dermestidae, two Bostrichidae, and five species of Ptinidae. The family Endecatomidae was reported for the first time for the province on the basis of *Endecatomus rugosus* (Randall). Here, three new Dermestidae, one new Bostrichidae, and 20 new Ptinidae are added to the faunal list for the province. One of these, *Ernobius opicus* Fall, is a new to Canada. Two of the three species of Dermestidae and all but one of the 20 species of Ptinidae were first detected using Lindgren funnel traps and nearly all specimens of these species were caught in these traps.

##### Family Dermestidae Latreille, 1804

###### Subfamily Megatominae Leach, 1815

####### Tribe Anthrenini Gistel, 1848

######## Anthrenus (Nathrenus) verbasci

Taxon classificationAnimaliaColeopteraDermestidae

(Linnaeus, 1767) †

######### Material examined.

**New Brunswick, York Co.**, Charters Settlement, 45.8395°N, 66.7391°W, 19.VI.2004, R.P. Webster // Mixed forest, on flowers of mountain ash (1, RWC).

######### Distribution in Canada and Alaska.

BC, AB, SK, MB, ON, QC, **NB**, NS (Bousquet et al. 2013).

###### Subfamily Megatominae Leach, 1815

####### Tribe Megatomini Leach, 1815

######## Megatoma (Perimegatoma) cylindrica

Taxon classificationAnimaliaColeopteraDermestidae

(Kirby, 1837)

######### Material examined.

**New Brunswick, Northumberland Co.**, ca, 2.5 km W of Sevogle, 47.0876°N, 65.8613°W, 1-14.V.2013, 11-25.VI.2014, C. Alderson & V. Webster // Old *Pinus banksiana* stand, Lindgren funnel traps (1, AFC; 2, RWC). **Gloucester Co.**, Bathurst, Daly Point Nature Preserve, 47.6392°N, 65.6098°W, 13-28.V.2015, 28.V-15.VI.2015, 25.VI-9.VII.2015, C. Alderson & V. Webster // Mixed forest, green Lindgren funnel traps in canopy of white pine (2), purple Lindgren funnel traps in canopy of white pine (1), purple Lindgren funnel trap in canopy (1) black Lindgren traps in canopy (2) (3, AFC; 3, RWC). **Restigouche Co.**, Dionne Brook P.N.A., 47.9030°N, 68.3503°W, 30.V-15.VI.2011, M. Roy & V. Webster // Old-growth northern hardwood forest, Lindgren funnel trap (1, AFC); ca. 3 km SE of Simpsons Field, 47.5277°N, 66.5142°W, 28.V-16.VI.2015, 25.VI-10.VII.2015, 10-23.VII.2015, C. Alderson & V. Webster // Old cedar & spruce forest with *Populus balsamifera* & *Populus tremuloides*, Lindgren funnel trap (4) in canopy of *Populus balsamifera* (2) (3, AFC; 3, RWC). **Sunbury Co.**, Acadia Research Forest, 45.9866°N, 66.3841°W, 19-25.V.2009, 25.V-2.VI.2009, R. Webster & M.-A. Giguère // Red spruce forest with red maple & balsam fir, Lindgren funnel traps (3, RWC).

######### Distribution in Canada and Alaska.

AK, YT, NT, BC, AB, ON, **NB** (Bousquet et al. 2013).

######## Trogoderma ornatum

Taxon classificationAnimaliaColeopteraDermestidae

(Say, 1825)

######### Material examined.

**New Brunswick, Queens Co.**, Jemseg, 45.8412°N, 66.1195°W, 10-25.VII.2012, C. Alderson, C. Hughes, & V. Webster // Hardwood woodland near seasonally flooded marsh, Lindgren funnel trap in canopy of *Quercus macrocarpa* (1, RWC); C.F.B. Gagetown, 45.7516°N, 66.1866°W, 3-15.VII.2013, 15-31.VII.2013, C. Alderson & V. Webster // Old mixed forest with *Quercus rubra*, Lindgren funnel traps in canopy of *Quercus rubra* (4, RWC).

######### Distribution in Canada and Alaska.

ON, QC, **NB**, NS (Bousquet et al. 2013).

##### Family Bostrichidae Latreille, 1802

###### Subfamily Lyctinae Billberg, 1820

####### Tribe Lyctini Billberg, 1820

######## Lyctus planicollis

Taxon classificationAnimaliaColeopteraBostrichidae

LeConte, 1858

######### Material examined.

**New Brunswick, Queens Co.**, Central Hampstead, 21.III.2008, Scott Makepeace // In house in empty aquarium (6, RWC).

######### Distribution in Canada and Alaska.

BC, MB, ON, QC, **NB**, NS, PE (Bousquet et al. 2013).

##### Family Ptinidae Latreille, 1802

###### Subfamily Eucradinae LeConte, 1861

####### Tribe Eucradini LeConte, 1861

######## Eucrada humeralis

Taxon classificationAnimaliaColeopteraPtinidae

(Melsheimer, 1846)

######### Material examined.

**New Brunswick, Carleton Co.**, Jackson Falls, “Bell Forest”, 46.2200°N, 67.7231°W, 7- 21.VI.2012, 21.VI-3.VII.2012, C. Alderson & V. Webster // Rich Appalachian hardwood forest, Lindgren funnel traps in canopy of *Fagus grandifolia* (2), *Fraxinus americana* (2), *Juglans cinerea* (4), and *Tilia americana* (1) (1, AFC; 8, RWC). **Queens Co.**, C.F.B. Gagetown, 45.7516°N, 66.1866°W, 4-17.VI.2013, 17.VI-3.VII.2013, 3-15.VII.2013, C. Alderson & V. Webster // Old mixed forest with *Quercus rubra*, Lindgren funnel trap in canopy of *Quercus rubra* (3, AFC; 1, NBM). **York Co.**, Fredericton, Odell Park, 45.9571°N, 66.6650°W, 28.VI-10.VII.2012, C. Alderson & V. Webster // Old-growth eastern hemlock forest, Lindgren funnel trap in canopy of *Betula alleghaniensis* (1, RWC); Fredericton, Odell Park, 45.9539°N, 66.6666°W, 10-24.VI.2013, 24.VI-9.VII.2013, C. Alderson & V. Webster // Hardwood stand, Lindgren funnel traps in canopy (2, AFC; 1, NBM; 2, RWC); Keswick Ridge, 45.9962°N, 66.8781°W, 3-18.VII.2014, C. Alderson & V. Webster // Mixed forest, Lindgren funnel trap 1 m high under trees (1, AFC).

######### Distribution in Canada and Alaska.

ON, QC, **NB** (Bousquet et al. 2013).

######### Comments.

All but one specimen were captured in Lindgren funnel traps in the canopy of various tree species during 2012, 2013, and 2014. Interestingly, this species was not detected at the Bell Forest or other sites in NB prior to 2012, possibly because sampling was not done using Lindgren traps in the canopy of trees before 2012.

###### Subfamily Ernobiinae Pic, 1912

####### Ernobius filicornis

Taxon classificationAnimaliaColeopteraPtinidae

LeConte, 1879

######## Material examined.

**New Brunswick, York Co.**, 15 km W of Tracy off Rt. 645, 45.6848°N, 66.8821°W, 28.VI-7.VII.2009, R. Webster & M.-A. Giguère // Old red pine forest, Lindgren funnel trap (1, RWC); same locality data and forest type, 26.VI.2009, R.P. Webster // u.v. light trap (2, RWC); same locality data and forest type, 13-27.VII.2010, R. Webster & C. MacKay // Lindgren funnel trap (1, RWC); 16 km W of Tracy off Rt. 645, 45.6854°N, 66.8839°W, 11-25.VII.2014, C. Alderson & V. Webster // Old red pine forest, Lindgren funnel trap in canopy of red pine (1, RWC).

######## Distribution in Canada and Alaska.

**NB**, NS (Bousquet et al. 2013).

####### Ernobius luteipennis

Taxon classificationAnimaliaColeopteraPtinidae

LeConte, 1879

######## Material examined.

**New Brunswick, Kent Co.**, Kouchibouguac National Park, 46.8072°N, 64.9100°W, 27.V-12.VI.2015, 12-24.VI.2015, 7-22.VII.2015, C. Alderson & V. Webster // Jackpine forest, Lindgren funnel traps, 1 m high (1, AFC; 10, RWC).

######## Distribution in Canada and Alaska.

QC, **NB** (Bousquet et al. 2013).

####### Ernobius opicus

Taxon classificationAnimaliaColeopteraPtinidae

Fall, 1905

######## Material examined.

**Canada, New Brunswick, York Co.** New Maryland, Charters Settlement, 45.8395°N, 66.7391°W, 27.VI.2007, R.P. Webster // Mixed forest, m.v. light (1, RWC).

######## Distribution in Canada and Alaska.

**NB (New Canadian record).**

######## Comments.

*Ernobius opicus* is easily distinguished from its congeners by the combination of scabrous and opaque (conspicuously granulate) elytra being slightly less shiny than the prothorax, fifth segment of antennae being as long as the third, and the peculiar lateral sinuation of the anterior edge of the pronotum (Fall 1905). The species was described from specimens from MA and MI.

####### Ernobius schedli

Taxon classificationAnimaliaColeopteraPtinidae

W.J. Brown, 1932

######## Material examined.

**New Brunswick, York Co.**, Canterbury, Eel River P.N.A., 45.8967°N, 67.6343°W, 8-21.V.2014, C. Alderson & V. Webster // Old-growth eastern white cedar swamp & fen, Lindgren funnel trap (1, RWC).

######## Distribution in Canada and Alaska.

ON, QC, **NB**, NS, NF (Bousquet et al. 2013).

####### Utobium elegans

Taxon classificationAnimaliaColeopteraPtinidae

(Horn, 1894)

######## Material examined.

**New Brunswick, Gloucester Co.**, Bathurst, Daly Point Nature Preserve, 47.6392°N, 65.6098°W, 13-28.V.2015, 25.VI-9.VII.2015, C. Alderson & V. Webster // Mixed forest, Lindgren funnel traps in canopy (2, RWC). **Northumberland Co.**, ca, 2.5 km W of Sevogle, 47.0876°N, 65.8613°W, 11-26.VI.2013, 26.VI-8.VII.2013, C. Alderson & V. Webster // Old *Pinus banksiana* stand, Lindgren funnel traps (2, RWC). **Restigouche Co.**, Dionne Brook P.N.A., 47.9030°N, 68.3503°W, 15-27.VI.2011, M. Roy & V. Webster // Old-growth northern hardwood forest, Lindgren funnel trap (1, RWC); ca. 3 km SE of Simpsons Field, 47.5277°N, 66.5142°W, 16-25.VI.2015, C. Alderson & V. Webster // Old cedar & spruce forest with *Populus balsamifera* & *Populus tremuloides*, Lindgren funnel trap (1, AFC). **York Co.**, 14 km WSW of Tracy, S of Rt. 645, 45.6741°N, 66.8661°W, 2-16.VI.2010, R. Webster & C. MacKay // Old mixed forest with red & white spruce, red and white pine, balsam fir, eastern white cedar, red maple & *Populus* sp., Lindgren funnel trap (1, RWC); Keswick Ridge, 45.9962°N, 66.8781°W, 4-19.VI.2014, C. Alderson & V. Webster // Mixed forest, Lindgren funnel trap in canopy (1, RWC).

######## Distribution in Canada and Alaska.

AK, YT, BC, AB, SK, MB, ON, QC, **NB** (Bousquet et al. 2013).

####### Utobium granulatum

Taxon classificationAnimaliaColeopteraPtinidae

R.E. White, 1976

######## Material examined.

**New Brunswick, Gloucester Co.**, Bathurst, Daly Point Nature Preserve, 47.6392°N, 65.6098°W, 13-28.V.2015, 25.VI-9.VII.2015, C. Alderson & V. Webster // Mixed forest, Lindgren funnel traps in canopy white pine (2, RWC).

######## Distribution in Canada and Alaska.

BC, AB, QC, **NB** (Bousquet et al. 2013).

####### Xestobium gaspensis

Taxon classificationAnimaliaColeopteraPtinidae

R. E. White, 1975

######## Material examined.

**New Brunswick, Charlotte Co.**, 10 km NW of New River Beach, 45.2110°N, 66.6170°W, 17-31.V.2010, R. Webster & C. MacKay // Old-growth eastern white cedar forest, Lindgren funnel trap (1, RWC). **Northumberland Co.**, Upper Graham Plains, 47.1001°N, 66.8154°W, 28.V-10.VI.2014, 10-24.VI.2014, C. Alderson & V. Webster // Old black spruce forest, Lindgren funnel traps (5, AFC; 2, NBM). **Restigouche Co.**, Dionne Brook P.N.A., 47.9064°N, 68.3441°W, 31.V-15.VI.2011, 15-27.VI.2011, M. Roy & V. Webster // Old-growth white spruce & balsam fir forest, Lindgren funnel traps (1, AFC; 1, NBM; 6, RWC); same locality and collectors but 47.9030°N, 68.3503°W, 30.V-15.VI.2011 // Old-growth northern hardwood forest, Lindgren funnel trap (1, RWC); ca. 3 km SE of Simpsons Field, 47.5277°N, 66.5142°W, 14-28.V.2015, 28.V-16.VI.2015, C. Alderson & V. Webster // Old cedar & spruce forest with *Populus balsamifera* & *Populus tremuloides*, Lindgren funnel traps (2, AFC). **York Co.**, Eel River P.N.A., 45.8966°N, 67.6345°W, 21.V-2.VI.2014, 2-20.VI.2014, C. Alderson & V. Webster // Old-growth eastern white cedar swamp & fen, Lindgren funnel trap (3, AFC; 2, NBM; 1, RWC).

######## Distribution in Canada and Alaska.

ON, QC, **NB**, NS (Bousquet et al. 2013).

###### Subfamily Anobiinae Fleming, 1821

####### Hemicoelus defectus

Taxon classificationAnimaliaColeopteraPtinidae

(Fall, 1905)

######## Material examined.

**New Brunswick, Kent Co.**, Kouchibouguac National Park, 46.8072°N, 64.9100°W, 20-31.VIII.2015, C. Alderson & V. Webster // Jackpine forest, Lindgren funnel trap, 1 m high (1, RWC). **Queens Co.**, Grand Lake Meadows P.N.A., 45.8227°N, 66.1209°W, 5-19.VII.2011, M. Roy & V. Webster // Old silver maple forest with green ash and seasonally flooded marsh, Lindgren funnel traps (2, RWC). **Sunbury Co.**, Gilbert Island, 45.8770°N, 66.2954°W, 12-29.VI.2012, 29.VI-11.VII.2012, C. Alderson, C. Hughes, & V. Webster // Hardwood forest, Lindgren funnel trap 1 m high under *Tilia americana* (2, RWC); Sunpoke Lake, 45.7656°N, 66.5550°W, 18.VI-9.VII.2012, C. Alderson & V. Webster // Red oak forest near seasonally flooded marsh, Lindgren funnel trap 1 m high under *Quercus rubra* (1, RWC). **York Co.**, Charters Settlement, 45.8395°N, 66.7391°W, 10.VI.2007, 25.VI.2009, R.P. Webster // Mixed forest, u.v. light (2, RWC); Keswick Ridge, 45.9962°N, 66.8781°W, 30.VI-16.VII.2015, C. Alderson & V. Webster // Hardwood forest, green Lindgren funnel traps 1 m high (2, RWC).

######## Distribution in Canada and Alaska.

BC, MB, ON, QC, **NB**, PE (Bousquet et al. 2013).

####### Hemicoelus pusillus

Taxon classificationAnimaliaColeopteraPtinidae

(Fall, 1905)

######## Material examined.

**New Brunswick, Carleton Co.**, Jackson Falls, “Bell Forest”, 46.2200°N, 67.7231°W, 25.VII.2007, R.P. Webster // Rich Appalachian hardwood forest, m.v. light (1, RWC). **Northumberland Co.**, ca. 1.5 km NW of Sevogle, 47.0939°N, 65.8387°W, 22.VII-6.VIII.2013, C. Alderson & V. Webster // *Populus tremuloides* stand with a few conifers, Lindgren funnel trap 1 m high under *Populus tremuloides* (1, RWC). **Queens Co.**, Cranberry Lake P.N.A., 46.1125°N, 65.6075°W, 13-20.VII.2011, M. Roy & V. Webster // Old red oak forest, Lindgren funnel trap (1, RWC); Jemseg, 45.8412°N, 66.1195°W, 8-21.VIII.2012, C. Alderson, C. Hughes, & V. Webster // Hardwood woodland near seasonally flooded marsh, Lindgren funnel trap in canopy of *Quercus macrocarpa* (1, RWC); C.F.B. Gagetown, 45.7516°N, 66.1866°W, 3-15.VII.2013, 15-31.VII.2013, 12-28.VIII.2013, C. Alderson & V. Webster // Old mixed forest with *Quercus rubra*, Lindgren funnel traps in canopy of *Quercus rubra* (2, AFC; 1, RWC). **Restigouche Co.**, Dionne Brook P.N.A., 47.9030°N, 68.3503°W, 28.VII-9.VIII.2011, M. Roy & V. Webster // Old-growth northern hardwood forest, Lindgren funnel trap (1, RWC). **Sunbury Co.**, Burton, near Sunpoke Lake, 45.7658°N, 66.5546°W, 27.VII.2007, R.P. Webster // Red oak & red maple forest, m.v. light (1, RWC). **York Co.**, 15 km W of Tracy off Rt. 645, 45.6848°N, 66.8821°W, 30.VI-13.VII.2010, R. Webster & K. Burgess // Old red pine forest, Lindgren funnel trap (1, RWC); Fredericton, Odell Park, 45.9539°N, 66.6666°W, 9-24.VII.2013, C. Alderson & V. Webster // Hardwood stand, Lindgren funnel 1 m high under trees (1, AFC; 1, RWC); Fredericton, U.N.B. Woodlot, 45.9206°N, 66.6520°W, 10-25.VII.2013, C. Alderson & V. Webster // Mature mixed forest, Lindgren funnel trap 5 m high (1, RWC); Keswick Ridge, 45.9962°N, 66.8781°W, 3-18.VII.2014, 18-30.VII.2014, 30.VII-13.VIII.2014, C. Alderson & V. Webster // Mixed forest, Lindgren funnel traps in canopy (1, AFC; 3, NBM).

######## Distribution in Canada and Alaska.

ON, QC, **NB** (Bousquet et al. 2013).

####### Oligomerus alternans

Taxon classificationAnimaliaColeopteraPtinidae

LeConte, 1865

######## Material examined.

**New Brunswick, Carleton Co.**, Meduxnekeag Valley Nature Preserve, 46.1957°N, 67.6803°W, 22.VII.2004, J. Edsall & R. Webster // Mixed forest, u.v. light trap (1, NBM; 1, RWC); Jackson Falls, “Bell Forest”, 46.2200°N, 67.7231°W, 22.VII.2004, K. Bredin, J. Edsall, & R. Webster // Rich Appalachian hardwood forest, u.v. light trap (1, NBM; 1, RWC); same locality data and forest type, 12.VII.2006, 25.VII.2007, 8.VII.2008, R.P. Webster // m.v. light (1, NBM; 3, RWC); same locality data and forest type, 3-17.VII.2012, 17-31.VII.2012, C. Alderson & V. Webster // Lindgren funnel traps in canopy of *Acer saccharum* (1), *Fagus grandifolia* (1), *Fraxinus americana* (1), *Juglans cinerea* (1), and *Tilia americana* (1) (2, AFC; 3, RWC). **Queens Co.**, Grand Lake Meadows P.N.A., 45.8227°N, 66.1209°W, 19.VII-5.VIII.2011, M. Roy & V. Webster // Silver maple swamp and seasonally flooded marsh, Lindgren funnel trap in forest canopy (2, RWC); C.F.B. Gagetown, 45.7516°N, 66.1866°W, 3-15.VII.2013, C. Alderson & V. Webster // Old mixed forest with *Quercus rubra*, Lindgren funnel trap in canopy of *Quercus rubra* (1, AFC). **Sunbury Co.**, Gilbert Island, 45.8770°N, 66.2954°W, 5-17.VII.2013, C. Alderson, C. Hughes & V. Webster (1, RWC). **York Co.**, Fredericton, Odell Park, 45.9484°N, 66.6802°W, 1-15.VIII.2014, C. Alderson & V. Webster // Old mixed forest, Lindgren funnel trap in canopy of tree (1, NBM); Keswick Ridge, 45.9962°N, 66.8781°W, 18-30.VII.2014, C. Alderson & V. Webster // Mixed forest, Lindgren funnel trap in canopy (1, NBM).

######## Distribution in Canada and Alaska.

ON, QC, **NB** (Bousquet et al. 2013).

####### Oligomerus obtusus

Taxon classificationAnimaliaColeopteraPtinidae

LeConte, 1865

######## Material examined.

**New Brunswick, Carleton Co.**, Jackson Falls, “Bell Forest”, 46.2200°N, 67.7231°W, 12.VII.2006, 25.VII.2007, R.P. Webster // Rich Appalachian hardwood forest, m.v. light (3, NBM; 1, RWC); same locality data, forest type, and collector, 12-19.VI.2008 // Lindgren funnel trap (1, RWC); same locality data and forest type, 3-17.VII.2012, 17-31.VII.2012, C. Alderson & V. Webster // Lindgren funnel traps in canopy of *Acer saccharum* (4), *Fagus grandifolia* (8), *Fraxinus americana* (2), *Juglans cinerea* (2), and *Tilia americana* (2) (7, AFC; 2, CNC; 2, NBM; 7, RWC). **Queens Co.**, C.F.B. Gagetown, 45.7516°N, 66.1866°W, 3-15.VII.2013, 15-31.VII.2013, 31.VII-12.VIII.2013, 12-28.VIII.2013, C. Alderson & V. Webster // Old mixed forest with *Quercus rubra*, Lindgren funnel trap in canopy of *Quercus rubra* (2, AFC; 3, NBM; 2, RWC). **York Co.**, Fredericton, Odell Park, 45.9571°N, 66.6650°W, 10-26.VII.2012, C. Alderson & V. Webster // Old-growth eastern hemlock forest, Lindgren funnel trap in canopy of *Tsuga canadensis* (1, AFC); Fredericton, Odell Park, 45.9539°N, 66.6666°W, 24.VII-7.VIII.2013, C. Alderson & V. Webster // Hardwood stand, Lindgren funnel traps in canopy (1, AFC; 1, NBM); Douglas, Currie Mountain, 45.9844°N, 66.7592°W, 24.VII-7.VIII.2013, C. Alderson & V. Webster // Mixed forest with *Quercus rubra*, Lindgren funnel trap in canopy of *Quercus rubra* (1, AFC); Fredericton, U.N.B. Woodlot, 45.9206°N, 66.6520°W, 10-25.VII.2013, C. Alderson & V. Webster // Mature mixed forest, Lindgren funnel trap 5 m high (1, AFC).

######## Distribution in Canada and Alaska.

ON, QC, **NB** (Bousquet et al. 2013).

######## Comment.

Most specimens (20 out of 26) of *Oligomerus obtusus* from NB were captured in Lindgren funnel traps in the canopy of various tree species in hardwood and mixed forests.

####### Platybregmus canadensis

Taxon classificationAnimaliaColeopteraPtinidae

Fisher, 1934

######## Material examined.

**New Brunswick**, Fredericton, Odell Park, 45.9539°N, 66.6666°W, 27.V-10.VI.2013, C. Alderson & V. Webster // Hardwood stand, Lindgren funnel trap 1 m high under trees (1, RWC).

######## Distribution in Canada and Alaska.

ON, **NB**, NS (Bousquet et al. 2013).

###### Subfamily Ptilininae Shuckard, 1839

####### Ptilinus pruinosus

Taxon classificationAnimaliaColeopteraPtinidae

Casey, 1898

######## Material examined.

**New Brunswick, Gloucester Co.**, Bathurst, Daly Point Nature Preserve, 47.6392°N, 65.6098°W, 25.VI-9.VII.2015, C. Alderson & V. Webster // Mixed forest, purple Lindgren funnel trap in canopy (2, AFC). **Northumberland Co.**, ca. 1.5 km NW of Sevogle, 47.0939°N, 65.8387°W, 11-26.VI.2013, 26.VI-8.VII.2013, C. Alderson & V. Webster // *Populus tremuloides* stand with a few conifers, Lindgren funnel trap in canopy of *Populus tremuloides* (4, AFC; 10, RWC). **York Co.**, Keswick Ridge, 45.9962°N, 66.8781°W, 19.VI-3.VII.2014, C. Alderson & V. Webster // Mixed forest, Lindgren funnel trap 1 m high under trees (1, AFC).

######## Distribution in Canada and Alaska.

MB, ON, QC, **NB**, NS (Bousquet et al. 2013).

######## Comments.

All but one of the 17 individuals of this species were captured in Lindgren funnel traps in the canopy of trees, most in *Populus tremuloides*.

###### Subfamily Xyletininae Gistel, 1848

####### Tribe Xyletinini Gistel, 1848

######## Vrilletta laurentina

Taxon classificationAnimaliaColeopteraPtinidae

Fall, 1905

######### Material examined.

**New Brunswick, Carleton Co.**, Jackson Falls, “Bell Forest”, 46.2200°N, 67.7231°W, 8-23.V.2012, 23.V-7.VI.2012, C. Alderson & V. Webster // Rich Appalachian hardwood forest, Lindgren funnel traps in canopy of *Tilia americana* (2, RWC); same locality and habitat data but 13-25.IV.2012, R. Webster, J. Sweeney & C. Hughes // Lindgren funnel trap in canopy of *Juglans cinerea* (1, AFC).

######### Distribution in Canada and Alaska.

ON, QC, **NB**, NS (Bousquet et al. 2013).

######## Xyletinus lugubris

Taxon classificationAnimaliaColeopteraPtinidae

LeConte, 1878

######### Material examined.

**New Brunswick, Carleton Co.**, Jackson Falls, “Bell Forest”, 46.2200°N, 67.7231°W, 23.V-7.VI.2012, 7-21.VI.2012, 21.VI-3.VII.2012, C. Alderson & V. Webster // Rich Appalachian hardwood forest, Lindgren funnel traps in canopy of *Fraxinus americana* (1) and *Tilia americana* (4) (2, AFC; 1, NBM; 3, RWC). **Gloucester Co.**, Bathurst, Daly Point Nature Preserve, 47.6392°N, 65.6098°W, 25.VI-9.VII.2015, 25.VI-9.VII.2015, C. Alderson & V. Webster // Mixed forest, Lindgren funnel trap in canopy (1, AFC). **Kent Co.**, Kouchibouguac National Park, 46.8087°N, 64.9078°W, 7-22.VII.2015, C. Alderson & V. Webster // Poplar/red maple stand, Lindgren funnel trap, 5 m high (1, AFC). **Northumberland Co.**, ca. 1.5 km NW of Sevogle, 47.0939°N, 65.8387°W, 8-22.VII.2013, C. Alderson & V. Webster // *Populus tremuloides* stand with a few conifers, Lindgren funnel trap in canopy of *Populus tremuloides* (1, RWC). **Queens Co.**, Jemseg, 45.8412°N, 66.1195°W, 12-28.VI.2012, C. Alderson, C. Hughes, & V. Webster // Hardwood woodland near seasonally flooded marsh, Lindgren funnel trap in canopy of *Quercus macrocarpa* (1, RWC). **Restigouche Co.**, Jacquet River Gorge P.N.A., 47.8257°N, 66.0764°W, 10-25.VI.2014, 25.VI-9.VII.2014, 9-22.VII.2014, C. Alderson & V. Webster // Old *Populus balsamifera* stand near river, Lindgren funnel trap in canopy of *Populus balsamifera* (1, AFC; 1, NBM; 2, RWC); ca. 3 km SE of Simpsons Field, 47.5277°N, 66.5142°W, 25.VI-10.VII.2015, C. Alderson & V. Webster // Old cedar & spruce forest with *Populus balsamifera* & *Populus tremuloides*, Lindgren funnel trap in canopy of *Populus balsamifera* (1, AFC). **Sunbury Co.**, Gilbert Island, 45.8770°N, 66.2954°W, 28.V-12.VI.2012, 12-29.VI.2012, 29.VI-11.VII.2012, 11-25.VII.2012, 6-20.VI.2013, C. Alderson, C. Hughes, & V. Webster // Hardwood forest, Lindgren funnel traps in canopy of *Juglans cinerea* (2), *Populus tremuloides* (1), and *Tilia americana* (3) (2, AFC; 1, NBM; 6, RWC).

######### Distribution in Canada and Alaska.

AB, SK, MB, ON, QC, **NB** (Bousquet et al. 2013).

######### Comments.

All but one of the 23 individuals of *Xyletinus lugubris* LeConte were captured in Lindgren funnel traps in the canopy of trees; one adult was captured at mid crown.

###### Subfamily Dorcatominae C. G. Thomson, 1859

####### Caenocara oculatum

Taxon classificationAnimaliaColeopteraPtinidae

(Say, 1824)

######## Material examined.

**New Brunswick, Queens Co.**, Cranberry Lake P.N.A., 46.1125°N, 65.6075°W, 18-31.VIII.2011, C. Hughes & R. Webster // Old red oak forest, Lindgren funnel trap (1, RWC); Jemseg, 45.8412°N, 66.1195°W, 10-25.VII.2012, 8-21.VIII.2012, C. Alderson, C. Hughes, & V. Webster // Hardwood woodland near seasonally flooded marsh, Lindgren funnel trap in canopy of *Quercus macrocarpa* (1, RWC). **York Co.**, Fredericton, 5-9.VI.2011, 12.V.2011, C.I.G. Adam // on foliage of birch (5, RWC).

######## Distribution in Canada and Alaska.

ON, QC, **NB**, NS, PE (Bousquet et al. 2013).

####### Dorcatoma falli

Taxon classificationAnimaliaColeopteraPtinidae

R.E. White, 1965

######## Material examined.

**New Brunswick, Carleton Co.**, Meduxnekeag Valley Nature Preserve, 46.1957°N, 67.6803°W, 28.VI.2005, R.P. Webster // Mixed forest, u.v. light trap (1, RWC). **Queens Co.**, Jemseg, 45.8412°N, 66.1195°W, 12-28.VI.2012, C. Alderson, C. Hughes, & V. Webster // Hardwood woodland near seasonally flooded marsh, Lindgren funnel trap 1 m high under *Quercus macrocarpa* (1, NBM; 8, RWC); C.F.B. Gagetown, 45.7516°N, 66.1866°W, 3-15.VII.2013, C. Alderson & V. Webster // Old mixed forest with *Quercus rubra*, Lindgren funnel trap in canopy of *Quercus rubra* (1, RWC). **Sunbury Co.**, Sunpoke Lake, 45.7656°N, 66.5550°W, 9-20.VII.2012, 3-15.VIII.2012, C. Alderson & V. Webster // Red oak forest near seasonally flooded marsh, Lindgren funnel traps in canopy of *Quercus rubra* (1), and 1 m high under *Quercus rubra* (1) (1, AFC; 1, RWC).

######## Distribution in Canada and Alaska.

ON, QC, **NB**, NS (Bousquet et al. 2013).

####### Sculptotheca puberula

Taxon classificationAnimaliaColeopteraPtinidae

(LeConte, 1865)

######## Material examined.

**New Brunswick, Carleton Co.**, Jackson Falls, “Bell Forest”, 46.2200°N, 67.7231°W, 12-19.VII.2008, R.P. Webster // Rich Appalachian hardwood forest, Lindgren funnel trap (1, RWC); same locality data and forest type, 19-31.VII.2009, R.P. Webster & M.-A. Giguère // Lindgren funnel trap (1, RWC). **Kent Co.**, Kouchibouguac National Park, 46.8072°N, 64.9100°W, 22.VII-4.VIII.2015, C. Alderson & V. Webster // Jackpine forest, Lindgren funnel trap, 1 m high (1, AFC). **Restigouche Co.**, ca. 3 km SE of Simpsons Field, 47.5277°N, 66.5142°W, 5-21.VIII.2015, C. Alderson & V. Webster // Old cedar & spruce forest with *Populus balsamifera* & *Populus tremuloides*, Lindgren funnel trap (1, AFC). **Queens Co.**, Cranberry Lake P.N.A., 46.1125°N, 65.6075°W, 10-15.VII.2009, 15-21.VII.2009, 21-28.VII.2009, 28.VII-6.VIII.2009, R.P. Webster & M.-A. Giguère // Old red oak forest, Lindgren funnel traps (8, RWC); C.F.B. Gagetown, 45.7516°N, 66.1866°W, 15-31.VII.2013, C. Alderson & V. Webster // Old mixed forest with *Quercus rubra*, Lindgren funnel trap in canopy of *Quercus rubra* (1, AFC). **Sunbury Co.**, Gilbert Island, 45.8770°N, 66.2954°W, 25.VII-8.VIII.2012, C. Alderson, C. Hughes, & V. Webster // Hardwood forest, Lindgren funnel trap in canopy of *Juglans cinerea* (1, AFC). **York Co.**, Douglas, Currie Mountain, 45.9832°N, 66.7564°W, 24.VI-9.VII.2013, 9-24.VII.2013, C. Alderson & V. Webster // Old *Pinus strobus* stand, Lindgren funnel traps in canopy of *Pinus strobus* (2, NBM); Fredericton, Odell Park, 45.9539°N, 66.6666°W, 9-24.VII.2013, C. Alderson & V. Webster // Hardwood stand, Lindgren funnel traps in canopy (1, AFC); Canterbury, Eel River P.N.A., 45.8966°N, 67.6345°W, 15-28.VII.2014, C. Alderson & V. Webster // Old-growth eastern white cedar swamp & fen, Lindgren funnel trap (1, NBM); Keswick Ridge, 45.9962°N, 66.8781°W, 3-18.VII.2014, 18-30.VII.2014, C. Alderson & V. Webster // Mixed forest, Lindgren funnel traps 1 m high under trees (2, AFC).

######## Distribution in Canada and Alaska.

ON, QC, **NB**, NS (Bousquet et al. 2013).

####### Stagetus profundus

Taxon classificationAnimaliaColeopteraPtinidae

(LeConte, 1865)

######## Material examined.

**New Brunswick, Kent Co.**, Kouchibouguac National Park, 46.8072°N, 64.9100°W, 7-22.VII.2015, C. Alderson & V. Webster // Jackpine forest, Lindgren funnel trap, 1 m high (1, AFC). **Northumberland Co.**, ca, 2.5 km W of Sevogle, 47.0876°N, 65.8613°W, 28.V-11.VI.2013, 11-26.VI.2013, C. Alderson & V. Webster // Old *Pinus banksiana* stand, Lindgren funnel traps (4, AFC; 1, NBM; 11, RWC).

######## Distribution in Canada and Alaska.

BC, ON, QC, **NB**, NS (Bousquet et al. 2013).

#### Superfamily Cleroidea Latreille, 1802

##### Family Cleridae Latreille, 1802

The Cleridae of NB were reviewed by Webster et al. (2012j). They newly recorded *Cymatodera bicolor* (Say) and added many additional records of *Zenodosus sanquineus* (Say). Here, we add four more species to the faunal list of the province. Nearly all specimens were captured in Lindgren funnel traps.

###### Subfamily Tillinae Fischer von Waldheim, 1813

####### Cymatodes inornata

Taxon classificationAnimaliaColeopteraCleridae

(Say, 1835)

######## Material examined.

**New Brunswick, Sunbury Co.**, Gilbert Island, 45.8770°N, 66.2954°W, 28.V-12.VI.2012, 12-29.VI.2012, 29.VI-11.VII.2012, 11-25.VII.2012, 25.VII-8.VIII.2012, C. Alderson, C. Hughes, & V. Webster // hardwood forest, Lindgren funnel traps in canopy of *Juglans cinerea* (3), *Tilia americana* (17), and *Populus tremuloides* (1) (11, AFC; 1, CNC; 2, NBM; 10, RWC).

######## Distribution in Canada and Alaska.

ON, QC, **NB** (Bousquet et al. 2013).

######## Comments.

All specimens of *Cymatodes inornata* (Say) were captured in Lindgren traps in the canopy of trees, none in traps 1 m above the forest floor, suggesting that this species may be a canopy specialist.

###### Subfamily Clerinae Latreille, 1802

####### Thanasimus trifasciatus

Taxon classificationAnimaliaColeopteraCleridae

(Say, 1825)

######## Material examined.

**New Brunswick, Northumberland Co.**, Upper Graham Plains, 47.1001°N, 66.8154°W, 24.VI-9.VII.2014, C. Alderson & V. Webster // Old black spruce forest with white pine, Lindgren funnel trap in canopy of white pine (1, RWC).

######## Distribution in Canada and Alaska.

ON, QC, **NB** (Bousquet et al. 2013).

####### Thanasimus undatulus undatulus

Taxon classificationAnimaliaColeopteraCleridae

(Say, 1835)

######## Material examined.

**New Brunswick, Gloucester Co.**, Bathurst, Daly Point Nature Preserve, 47.6392°N, 65.6098°W, 13-28.V.2015, 28.V-15.VI.2015, C. Alderson & V. Webster // Mixed forest, black Lindgren funnel trap in canopy (1), 1 m high under trees (1) (2, AFC). **Kent Co.**, Kouchibouguac National Park, 46.8072°N, 64.9100°W, 21-27V.2015, C. Alderson & V. Webster // Jackpine forest, Lindgren funnel traps, 1 m high (2, AFC). **Northumberland Co.**, ca, 2.5 km W of Sevogle, 47.0876°N, 65.8613°W, 1-14.V.2013, 6-21.VIII.2013, C. Alderson & V. Webster // Old *Pinus banksiana* stand, Lindgren funnel traps (2, AFC); ca. 1.5 km NW of Sevogle, 47.0939°N, 65.8387°W, 11-26.VI.2013, C. Alderson & V. Webster // *Populus tremuloides* stand with a few conifers, Lindgren funnel traps in canopy of *Populus tremuloides* (1, AFC); Upper Graham Plains, 47.1001°N, 66.8154°W, 28.V-10.VI.2014, 24.VI-9.VII.2014, C. Alderson & V. Webster // Old black spruce forest, Lindgren funnel traps (1, AFC, 1, NBM). **Restigouche Co.**, Jacquet River Gorge P.N.A., 47.8257°N, 66.0764°W, 15-29.V.2014, C. Alderson & V. Webster // Old *Populus balsamifera* stand near river, Lindgren funnel trap 1 m high under trees (1, NBM). **Sunbury Co.**, Acadia Research Forest, 45.9866°N, 66.3441°W, 13-19.V.2009, 19-25.V.2009, 2-9.VI.2009, 9-16.VI.2009, 16-24.VI.2009, R.P. Webster & M.-A. Giguère // Red spruce forest with red maple & balsam fir, Lindgren funnel traps (7, AFC; 4, RWC). **York Co.**, Fredericton, 17.VI.1929, M.L. Prebble (2, AFC); Charters Settlement, 45.8430°N, 66.7375°W, 11.VII.2005, R.P. Webster // Regenerating mixed forest, on spruce log (1, RWC); 15 km W of Tracy off Rt. 645, 45.6848°N, 66.8821°W, 19-25.V.2009, 15-21.VI.2009, 21-28.VI.2009, 7-14.VII.2009, R.P. Webster & M.-A. Giguère // Old red pine forest, Lindgren funnel traps (6, AFC; 2, RWC); same locality data and forest type but 13.V.2009, R.P. Webster // On small branches of recently fallen red pine (2, RWC); Canterbury, Eel River P.N.A., 45.8966°N, 67.6345°W, 8-21.V.2014, C. Alderson & V. Webster // Old-growth eastern white cedar swamp & fen, Lindgren funnel trap (1, NBM); Fredericton, Odell Park, 45.9484°N, 66.6802°W, 12-22.V.2014, C. Alderson & V. Webster // Old mixed forest, Lindgren funnel trap in canopy of conifer (1, AFC).

######## Distribution in Canada and Alaska.

AK, NT, BC, AB, ON, QC, **NB**, NS (Bousquet et al. 2013).

######## Comments.

Two subspecies of *Thanasimus undatulus* (Say), *Thanasimus undatulus undatulus* and *Thanasimus undatulus nubilus* (Klug), co-occur at many sites in NB. The two subspecies differ in overall coloration, color pattern, and size. *Thanasimus undatulus undatulus* is black with the basal third of pronotum red-brown, and the white posterior band on elytra continues as a narrow band along the suture to near or to the posterior margin of the elytra. The average length is smaller: from 5.2 to 6.8 mm. *Thanasimus undatulus nubilus* is black, without red-brown areas on the pronotum and elytra, and there is little or no extension of the white posterior band along the suture toward elytral apex (body length 6.2 to 9.3 mm). We have not seen any intermediate specimens. More studies are required to establish if these two subspecies should be treated as distinct species.

###### Subfamily Korynetinae Laporte, 1836

####### Chariessa pilosa

Taxon classificationAnimaliaColeopteraCleridae

(Forster, 1771)

######## Material examined.

**New Brunswick, Queens Co.**, Jemseg, 45.8412°N, 66.1195°W, 28.VI-10.VII.2012, 10-25.VII.2012, 25.VII-8.VIII.2012, 8-21.VIII.2012, C. Alderson, C. Hughes, & V. Webster // Hardwood woodland near seasonally flooded marsh, Lindgren funnel trap in canopy of *Quercus macrocarpa* (10, AFC; 1, CNC; 1, NBM; 6, RWC); C.F.B. Gagetown, 45.7516°N, 66.1866°W, 17.VI-3.VII.2013, 3-15.VII.2013, 15-31.VII.2013, 12-28.VIII.2013, C. Alderson & V. Webster // Old mixed forest with *Quercus rubra*, Lindgren funnel trap in canopy of *Quercus rubra* (4, AFC, 3, NBM, 2, RWC). **Sunbury Co.**, Sunpoke Lake, 45.7656°N, 66.5550°W, 9-20.VII. 2012, C. Alderson & V. Webster // Red oak forest near seasonally flooded marsh, Lindgren funnel trap in canopy of *Quercus rubra* (1, AFC); Gilbert Island, 45.8770°N, 66.2954°W, 12-29.VI.2012, 11-25.VII.2012, C. Alderson, C. Hughes, & V. Webster // hardwood forest, Lindgren funnel traps in canopy of *Juglans cinerea* (2, RWC). **York Co.**, Fredericton, Odell Park, 45.9539°N, 66.6666°W, 24.VI-9.VII.2013, 9-24.VII.2013, 7-19.VIII.2013, C. Alderson & V. Webster // Hardwood stand, Lindgren funnel traps in canopy (4, AFC; 2, NBM); Douglas, Currie Mountain, 45.9832°N, 66.7564°W, 9-24.VII.2013, C. Alderson & V. Webster // Old *Pinus strobus* stand, Lindgren funnel traps in canopy of *Pinus strobus* (1, AFC); Canterbury, Eel River P.N.A., 45.8966°N, 67.6345°W, 15-28.VII.2014, 28.VII-12.VIII.2014, C. Alderson & V. Webster // Old-growth eastern white cedar swamp & fen, Lindgren funnel traps (3, AFC, 1, NBM); 16 km W of Tracy off Rt. 645, 45.6854°N, 66.8839°W, 25.VII-8.VIII.2014, C. Alderson & V. Webster // Old red pine forest, Lindgren funnel trap in canopy of red pine (1, AFC); Keswick Ridge, 45.9962°N, 66.8781°W, 3-18.VII.2014, 18-30.VII.2014, C. Alderson & V. Webster // Mixed forest, Lindgren funnel traps in canopy (2, AFC; 2, NBM).

######## Distribution in Canada and Alaska.

BC, AB, SK, MB, ON, QC, **NB** (Bousquet et al. 2013).

######## Comments.

All specimens of *Chariessa pilosa* reported from NB were captured in Lindgren funnel traps. At sites (seven of eight sites where species was detected) where both high (canopy) and low traps were tested, *Chariessa pilosa* was captured exclusively in traps deployed in the canopy of trees. This species was captured in low traps at only one site (Eel River P.N.A.). At this site, canopy traps were not used, and the four specimens were captured in traps along the edge of an open fen.

##### Family Melyridae Leach, 1815

The Melyridae of NB were reviewed by Webster et al. (2012j). Two species, *Attalus morulus* (LeConte) and *Dolichosoma foveicolle* (Kirby), were newly reported for the province. Here, we add four more species to the faunal list of NB.

###### Subfamily Dasytinae Laporte, 1840

####### Tribe Dasytini Laporte, 1840

######## Hoppingiana hudsonica

Taxon classificationAnimaliaColeopteraMelyridae

(LeConte, 1866)

######### Material examined.

**New Brunswick, Northumberland Co.**, ca. 2.5 km W of Sevogle, 47.0876°N, 65.8613°W, 26.VI-8.VII.2013, C. Alderson & V. Webster // Old *Pinus banksiana* stand, Lindgren funnel traps (2, RWC).

######### Distribution in Canada and Alaska.

YT, NT, BC, AB, SK, MB, QC, **NB** (Bousquet et al. 2013).

###### Subfamily Malachiinae Fleming, 1821

####### Tribe Malachiini Fleming, 1821

######## Attalus (Acletus) nigrellus

Taxon classificationAnimaliaColeopteraMelyridae

(LeConte, 1852)

######### Material examined.

**New Brunswick, Northumberland Co.**, Upper Graham Plains, 47.1001°N, 66.8154°W, 10-25.VI.2014, C. Alderson & V. Webster // Old black spruce forest, Lindgren funnel trap (1, RWC); same locality and habitat data, and collection method but 24.VI-9.VII.2014, K. Dearborn & C. Hughes (1, RWC). **Restigouche Co.**, Jacquet River Gorge P.N.A., 47.8257°N, 66.0764°W, 10-25.VI.2014, C. Alderson & V. Webster // Old *Populus balsamifera* stand near river, Lindgren funnel trap in canopy of *Populus balsamifera* (1, RWC).

######### Distribution in Canada and Alaska.

BC, SK, ON, QC, **NB** (Bousquet et al. 2013).

######## Attalus (Attalus) terminalis

Taxon classificationAnimaliaColeopteraMelyridae

(Erichson, 1840)

######### Material examined.

**New Brunswick, Queens Co.**, Mount Douglas, 230 m elev., 45.4654°N, 66.3501°W, 4.VII.2012, R.P. Webster // Granitic bald, sweeping low shrubs on margin of bald (mostly *Vaccinium* & *Kalmia*) (3, RWC).

######### Distribution in Canada and Alaska.

MB, ON, QC, **NB**, NS (Bousquet et al. 2013).

######## Collops tricolor

Taxon classificationAnimaliaColeopteraMelyridae

(Say, 1823)

######### Material examined.

**New Brunswick, Queens Co.**, Mount Douglas, 230 m elev., 45.4654°N, 66.3501°W, 1.VII.2012, R.P. Webster, M.-A. Giguère & M. Lavoie // Granitic bald, on sun-exposed bare rock face (7, NBM; 8, RWC).

######### Distribution in Canada and Alaska.

SK, MB, ON, QC, **NB**, NS (Bousquet et al. 2013).

######### Comments.

This species was reported from dry lichen communities on granite outcrops on a lake margin in NS (Majka 2005). Specimens from NB were found on a granitic bald on a sun-exposed bare rock face. The bright-colored adults were observed crawling over the surface of the lichen-covered rockface in full sun.

#### Superfamily Cucujoidea Latreille, 1802

##### Family Monotomidae Laporte, 1840

Three species of Monotomidae were newly recorded for NB by Webster et al. (2012l). Here, we report another two species, which were detected using Lindgren funnel traps.

###### Subfamily Monotominae Laporte, 1840

####### Tribe Europini Sen Gupta, 1988

######## Bactridium striolatum

Taxon classificationAnimaliaColeopteraMonotomidae

(Reitter, 1873)

######### Material examined.

**New Brunswick, Carleton Co.**, Jackson Falls, “Bell Forest”, 46.2200°N, 67.7231°W, 23.V-7.VI.2012, C. Alderson & V. Webster // Rich Appalachian hardwood forest, Lindgren funnel trap in canopy of *Juglans cinerea* (1, RWC). **York Co.**, Keswick Ridge, 45.9962°N, 66.8781°W, 30.VI-16.VII.2015, C. Alderson & V. Webster // Mixed forest, Lindgren funnel trap 1 m high under trees (1, RWC).

######### Distribution in Canada and Alaska.

ON, QC, **NB** (Bousquet et al. 2013).

####### Tribe Monotomini Laporte, 1840

######## Monotoma americana

Taxon classificationAnimaliaColeopteraMonotomidae

Aubé, 1837

######### Material examined.

**New Brunswick, Northumberland Co.**, ca. 1.5 km NW of Sevogle, 47.0939°N, 65.8387°W, 1-14.V.2013, C. Alderson & V. Webster // *Populus tremuloides* stand with a few conifers, Lindgren funnel trap 1 m high under *Populus tremuloides* (1, RWC).

######### Distribution in Canada and Alaska.

ON, QC, **NB** (Bousquet et al. 2013).

##### Family Cryptophagidae Kirby, 1826

The Cryptophagidae of NB were reviewed by Webster et al. (2012l). Six species were newly recorded for the province, and the presence of *Antherophagus convexulus* LeConte was confirmed. Klimaszewski et al. (2015) newly recorded the adventive *Cryptophagus saginatus* Sturm and *Cryptophagus subfumatus* Kraatz in a review of the adventive Cucujoidea of Canada. Here, we newly record an additional 10 species from NB, many of which were captured in Lindgren funnel traps.

###### Subfamily Cryptophaginae Kirby, 1826

####### Tribe Caenoscelini Casey, 1900

######## Caenoscelis basalis

Taxon classificationAnimaliaColeopteraCryptophagidae

Casey, 1900

######### Material examined.

**New Brunswick, Carleton Co.**, Jackson Falls, “Bell Forest”, 46.2200°N, 67.7231°W, 4-12.VI.2008, R.P. Webster // Rich Appalachian hardwood forest with some conifers, Lindgren funnel trap (1, RWC); same data as before except 23-28.IV.2009, 8-16.VI.2009, R. Webster & M.-A. Giguère (2, RWC). **Queens Co.**, Grand Lake Meadows P.N.A., 45.8227°N, 66.1209°W, 19-31.V.2010, R. Webster & C. MacKay // Old silver maple forest with green ash and seasonally flooded marsh, Lindgren funnel trap (1, RWC). **York Co.**, Charters Settlement, 45.8395°N, 66.7391°W, 17.IV.2005, R.P. Webster // Mixed forest, in flight during warm sunny afternoon (2, RWC); same locality data and collector but 1.VIII.2004, m.v. light (1, RWC); same locality data and collector but 9.IV.2005 // Residential lawn among lawn grass (1, RWC); same locality and collector but 45.8456°N, 66.7267°W, 16.V.2010 // Beaver dam among sticks and debris near an overflow area of dam (1, RWC).

######### Distribution in Canada and Alaska.

ON, QC, **NB**, NS, NF (Bousquet et al. 2013).

######## Caenoscelis subdeplanata

Taxon classificationAnimaliaColeopteraCryptophagidae

Brisout de Barneville, 1882*

######### Material examined.

**New Brunswick, Northumberland Co.**, ca, 2.5 km W of Sevogle, 47.0876°N, 65.8613°W, 22.VII-6.VIII.2013, C. Alderson & V. Webster // Old *Pinus banksiana* stand, Lindgren funnel trap (1, RWC). **Restigouche Co.**, Dionne Brook P.N.A., 47.9064°N, 68.3441°W, 14-28.VII.2011, 28.VII-9.VIII.2011, M. Roy & V. Webster // Old-growth white spruce & balsam fir forest, Lindgren funnel traps (4, RWC); same locality and collectors but 47.9030°N, 68.3503°W, 27.VI-14.VII.2011 // Old-growth northern hardwood forest, Lindgren funnel trap (2, RWC).

######### Distribution in Canada and Alaska.

AB, MB, ON, QC, **NB** (Bousquet et al. 2013).

####### Tribe Cryptophagini Kirby, 1826

######## Cryptophagus corticinus

Taxon classificationAnimaliaColeopteraCryptophagidae

C.G. Thomson, 1863*

######### Material examined.

**New Brunswick, Kent Co.**, Kouchibouguac National Park, 46.8087°N, 64.9078°W, 27.V-12.VI.2015, C. Alderson & V. Webster // Poplar/red maple stand, Lindgren funnel trap, 1 m high (1, RWC). **Restigouche Co.**, Dionne Brook P.N.A., 47.9030°N, 68.3503°W, 15-27.VI.2011, M. Roy & V. Webster // Old-growth northern hardwood forest, Lindgren funnel trap (1, RWC). **Sunbury Co.**, Gilbert Island, 45.8770°N, 66.2954°W, 18-28.V.2012, C. Alderson, C. Hughes, & V. Webster // hardwood forest, Lindgren funnel trap 1 m high under *Tilia americana* (1, RWC).

######### Distribution in Canada and Alaska.

AK, BC, SK, ON, QC, **NB** (Bousquet et al. 2013).

######## Cryptophagus difficilis

Taxon classificationAnimaliaColeopteraCryptophagidae

Casey, 1900

######### Material examined.

**New Brunswick, Northumberland Co.**, ca. 2.5 km W of Sevogle, 47.0876°N, 65.8613°W, 21.VIII.2013, 27.VIII.2013, R.P. Webster // Old jack pine forest, in dried bolete mushrooms on roadside (2, AFC; 1, NBM; 11, RWC).

######### Distribution in Canada and Alaska.

BC, AB, SK, QC, **NB**, LB, NF (Bousquet et al. 2013).

######## Cryptophagus jakowlewi

Taxon classificationAnimaliaColeopteraCryptophagidae

Reitter, 1888*

######### Material examined.

**New Brunswick, Restigouche Co.**, Dionne Brook P.N.A., 47.9064°N, 68.3441°W, 31.V-15.VI.2011, 27.VI-14.VII.2011, 28.VII-9.VIII.2011, M. Roy & V. Webster // Old-growth white spruce & balsam fir forest, Lindgren funnel traps (9) and flight intercept traps (8) (7, AFC, 10, RWC); ca. 3 km SE of Simpsons Field, 47.5277°N, 66.5142°W, 14-28.V.2015, C. Alderson & V. Webster // Old cedar & spruce forest with *Populus balsamifera* & *Populus tremuloides*, Lindgren funnel trap (1, RWC).

######### Distribution in Canada and Alaska.

AK, NT, BC, AB, SK, QC, **NB**, NS, PE (Bousquet et al. 2013).

######## Cryptophagus scanicus

Taxon classificationAnimaliaColeopteraCryptophagidae

(Linnaeus, 1758)†

######### Material examined.

**New Brunswick, Queens Co.**, Jemseg, 45.8412°N, 66.1195°W, 21.VIII-7.IX.2012, C. C. Hughes, & K. Van Rooyen // Hardwood woodland near seasonally flooded marsh, Lindgren funnel trap 1 m high under *Quercus macrocarpa* and in canopy of *Quercus macrocarpa* (2, RWC); Grand Lake Meadows P.N.A., 45.8227°N, 66.1209°W, 24.VIII-3.IX.2010, R.P. Webster // Old silver maple forest with green ash and seasonally flooded marsh, Lindgren funnel trap (1, RWC). **York Co.**, Charters Settlement, 45.8395°N, 66.7391°W, 3.X.2007, R.P. Webster // Mixed forest, in decaying (moldy) corncobs & cornhusks (1, RWC).

######### Distribution in Canada and Alaska.

**NB**, NF (Bousquet et al. 2013).

######### Comments.

This adventive Palaearctic species was previously known only from NF (St. John’s) in North America and occurs in various habitats including stored produce in Europe (Woodroffe and Coombs 1961, Johnson et al. 2007, Klimaszewski et al. 2015). Specimens from NB were captured in Lindgren funnel traps and sifted from a pile of moldy decaying corncobs and cornhusks.

######## Cryptophagus scutellatus

Taxon classificationAnimaliaColeopteraCryptophagidae

Newman, 1834†

######### Material examined.

**New Brunswick, Gloucester Co.**, Bathurst, Daly Point Nature Preserve, 47.6392°N, 65.6098°W, 13-28.V.2015, C. Alderson & V. Webster // Mixed forest, black Lindgren funnel trap in canopy (1, RWC). **Kent Co.**, Kouchibouguac National Park, 46.8072°N, 64.9100°W, 27.V-24.VI.2015, C. Alderson & V. Webster // Jackpine forest, Lindgren funnel trap, 1 m high (1, RWC). **Restigouche Co.**, ca. 3 km SE of Simpsons Field, 47.5277°N, 66.5142°W, 14-28.V.2015, C. Alderson & V. Webster // Old cedar & spruce forest with *Populus balsamifera* & *Populus tremuloides*, Lindgren funnel trap (1, AFC). **York Co.**, Fredericton, Odell Park, 45.9571°N, 66.6650°W, 15.V-1.VI.2012, C. Alderson & V. Webster // Old-growth eastern hemlock forest, Lindgren funnel trap 1 m high under *Betula alleghaniensis* (1, RWC); Keswick Ridge, 45.9962°N, 66.8781°W, 5-19.V.2015, 19.V-3.VI.2015, 18-30.VI.2015, C. Alderson & V. Webster // Mixed forest, Lindgren funnel traps 1 m high under trees (4, RWC).

######### Distribution in Canada and Alaska.

ON, QC, **NB** (Bousquet et al. 2013, Klimaszewski et al. 2015).

######## Cryptophagus setulosus

Taxon classificationAnimaliaColeopteraCryptophagidae

Sturm, 1845*

######### Material examined.

**New Brunswick, York Co.**, Charters Settlement, 45.8395°N, 66.7391°W, 1.VIII.2004, R.P. Webster // Mixed forest, m.v. light (1, RWC).

######### Distribution in Canada and Alaska.

BC, SK, ON, QC, **NB**, LB, NF (Bousquet et al. 2013).

###### Subfamily Atomariinae LeConte, 1861

####### Tribe Atomariini LeConte, 1861

######## Atomaria (Anchicera) lederi

Taxon classificationAnimaliaColeopteraCryptophagidae

Johnson, 1970‡

######### Material examined.

**New Brunswick, Carleton Co.**, Meduxnekeag Valley Nature Preserve, 46.1907°N, 67.6740°W, 23.V-7.VI.2012, C. Alderson & V. Webster // Old mixed forest, Lindgren funnel trap 1 m high under *Populus tremuloides* (1, RWC). **Northumberland Co.**, ca, 2.5 km W of Sevogle, 47.0876°N, 65.8613°W, 1-14.V.2013, C. Alderson & V. Webster // Old *Pinus banksiana* stand, Lindgren funnel traps (1, AFC; 3, RWC); same locality, collectors, forest type, and trapping method but 47.0879°N, 65.8585°W, 13-27.V.2014 (1, AFC; 3, RWC). **Queens Co.**, Cranberry Lake P.N.A., 46.1125°N, 65.6075°W, 27.V-5.VI.2009, R.P. Webster & M.-A. Giguère // Old red oak forest, Lindgren funnel trap (1, RWC). **Restigouche Co.**, Jacquet River Gorge P.N.A., 47.8257°N, 66.0764°W, 15-29.V.2014, C. Alderson & V. Webster // Old *Populus balsamifera* stand near river, Lindgren funnel trap in canopy of *Populus balsamifera* (1, RWC); Dionne Brook P.N.A., 47.9064°N, 68.3441°W, 31.V-15.VI.2011, M. Roy & V. Webster // Old-growth white spruce & balsam fir forest, Lindgren funnel trap (1, RWC). **Sunbury Co.**, Acadia Research Forest, 45.9866°N, 66.3441°W, 9-16.VI.2009, R.P. Webster & M.-A. Giguère // Red spruce forest with red maple & balsam fir, Lindgren funnel trap (1, RWC).

######### Distribution in Canada and Alaska.

**NB**, NS (Bousquet et al. 2013).

######### Comments.

*Atomaria lederi* Johnson was first reported for North America by Johnson et al. (2007) without supporting data. Majka et al. (2010) provided supporting data for Johnson’s record and additional locality and habitat data from NS for the presence of *Atomaria lederi* in Canada and North America. This species is widespread in NS and was found mostly in red spruce (*Picea rubens* Sarg.) and red spruce–eastern hemlock (*Tsuga canadensis* (L.) Carr.) forests (Majka et al. 2010). New Brunswick specimens were collected from Lindgren funnel traps in a mixed forest, jack pine (*Pinus banksiana* Lamb.) forest, red oak (*Quercus rubra* L.) forest, old-growth white spruce (*Picea glauca* (Moench) Voss) and balsam fir (*Abies balsamea* (L.) Mill.) forest, and a red spruce forest with some red maple (*Acer rubrum* L.) and balsam fir. Bousquet et al. (2013) considered the status of *Atomaria lederi* in North America as uncertain and that the species could either be adventive to North America or Holarctic. Klimaszewski et al. (2015) treated *Atomaria lederi* as an adventive Palaearctic species. Given that this species was collected in natural habitats throughout NB and NS suggests it could be a Holarctic species that has been undetected in Canada and North America until recently.

######## Atomaria (Atomaria) nigrirostris

Taxon classificationAnimaliaColeopteraCryptophagidae

Stephens, 1830*

######### Material examined.

**New Brunswick, Restigouche Co.**, Jacquet River Gorge P.N.A., 47.7843°N, 65.9795°W, 13.VI.2009, R.P. Webster // Upland black spruce forest, sweeping foliage (1, RWC); Dionne Brook P.N.A., 47.9030°N, 68.3503°W, 30.V-15.VI.2011, M. Roy & V. Webster // Old-growth northern hardwood forest, Lindgren funnel trap (1, RWC). **Saint John Co.**, Dipper Harbour, 45.1182°N, 66.3790°W, 28.V.2010, R.P. Webster // Upper margin of salt marsh, in grass litter in seepage area with *Carex* & *Spartina patens* (1, RWC). **Sunbury Co.**, Acadia Research Forest, 45.9866°N, 66.3441°W, 19-25.V.2009, R.P. Webster & M.-A. Giguère // Red spruce forest with red maple & balsam fir, Lindgren funnel trap (1, RWC). **York Co.**, Charters Settlement, 45.8395°N, 66.7391°W, 1.VIII.2007, R.P. Webster // Mixed forest, m.v. light (1, RWC); same locality data and collector but 23.IV.2008, 6.V.2008, 2.V.2010, 17.V.2010, 3.V.2012 // Mixed forest opening, collected with net between 16:30 and 19:00h (2, NBM; 2, RWC); same locality data and collector but 21.IX.2007 // Mixed forest, decaying (moldy) corncobs & cornhusks (1, RWC); 15 km W of Tracy off Rt. 645, 45.6848°N, 66.8821°W, 19-25.V.2009, R.P. Webster & M.-A. Giguère // Old red pine forest, Lindgren funnel trap (1, RWC); Douglas, Currie Mountain, 45.9844°N, 66.7592°W, 3-15.V.2013, C. Alderson & V. Webster // Mixed forest with *Quercus rubra*, Lindgren funnel trap 1 m high under *Quercus rubra* (1, AFC).

######### Distribution in Canada and Alaska.

AK, QC, **NB**, NS, PE, LB, NF (Bousquet et al. 2013).

##### Family Silvanidae Kirby, 1837

The Silvanidae of NB were reviewed by Webster et al. (2012k). They newly reported *Silvanus muticus* Sharp and reinstated *Ahasverus longulus* (Blatchley) to the provincial list. Here, we newly report another two species.

###### Subfamily Brontinae Blanchard, 1845

####### Tribe Telephanini LeConte, 1861

######## Telephanus atricapillus

Taxon classificationAnimaliaColeopteraSilvanidae

Erichson, 1846

######### Material examined.

**New Brunswick, York Co.**, Keswick Ridge, 45.9962°N, 66.8781°W, 19.V-3.VI.2015, C. Alderson & V. Webster // Mixed forest, purple Lindgren funnel trap 1 m high (1, RWC).

######### Distribution in Canada and Alaska.

ON, QC, **NB** (Bousquet et al. 2013).

######### Comments.

This species is listed as *Telephanus velox* (Haldeman) by Bousquet et al. (2013). Thomas and Nearns (2008) treat *Telephanus velox* as a synonym of *Telephanus atricapillus* Erichson. This publication should be consulted for details.

###### Subfamily Silvaninae Kirby, 1837

####### Ahasverus advena

Taxon classificationAnimaliaColeopteraSilvanidae

(Waltl, 1834)†

######## Material examined.

**New Brunswick, York Co.**, Keswick Ridge, 45.9962°N, 66.8781°W, 29.VII-13.VIII.2015, 27.VIII-9.IX.2015, C. Alderson & V. Webster // Mixed forest, purple Lindgren funnel traps in canopy (2, RWC).

######## Distribution in Canada and Alaska.

BC, AB, SK, MB, ON, QC, **NB**, NS, PE (Bousquet et al. 2013).

######## Comments.

This adventive and cosmopolitan species is found in various stored products such as lima beans, pigeon peas, decaying soybeans, stored grains, fruit, nuts, damp flour, rice, and moldy grass and feeds on surface molds on these items (Campbell et al. 1989, Thomas 1993). The two specimens from NB were captured in Lindgren funnel traps in the canopy of trees in a mixed forest. This stored product pest is not normally associated with natural habitats. There are a number of farms with barns within 2 km of the site that could have been the source of these specimens.

##### Family Laemophloeidae Ganglbauer, 1899

The Laemophloeidae of NB were reviewed by Webster at al. (2012k). They reported five species new to the province. Here, we newly report the adventive *Cryptolestes turcicus* (Grouvelle).

###### Cryptolestes turcicus

Taxon classificationAnimaliaColeopteraLaemophloeidae

(Grouvelle, 1876)†

####### Material examined.

**New Brunswick, York Co.**, Douglas, Currie Mountain, 45.9832°N, 66.7564°W, 24.VI-9.VII.2013, C. Alderson & V. Webster // Old *Pinus strobus* stand, Lindgren funnel traps in canopy of *Pinus strobus* (3), 1 m high under trees (2) (5, RWC); Douglas, Currie Mountain, 45.9844°N, 66.7592°W, 24.VI-9.VII.2013, 9-24.VII.2013, C. Alderson & V. Webster // Mixed forest with *Quercus rubra*, Lindgren funnel trap in canopy of *Quercus rubra* (1), 1 m high under trees (3) [1 male dissected] (4, RWC); Keswick Ridge, 45.9962°N, 66.8781°W, 19.VI-3.VII.2014, 13-29.VIII.2014, C. Alderson & V. Webster // Mixed forest, Lindgren funnel traps in canopy (1), 1 m high under trees (1) (2, RWC).

####### Distribution in Canada and Alaska.

BC, AB, SK, MB, ON, QC, **NB**, NS (Bousquet et al. 2013).

####### Comments.

The adventive *Cryptolestes turcicus* is considered a serious pest of flour and feed mills and is sometimes found in grain elevators and warehouses (Bousquet 1990). Specimens from NB were captured in Lindgren funnel traps in an old white pine (*Pinus strobus* L.) stand, mixed forest with red oak, and a mixed forest. This stored product pest is not normally associated with natural habitats. Several poultry farms occur in the vicinity of the sites, and it is likely that these were dispersing individuals from these farms. Interestingly, two other stored product pests, *Cryptolestes pusillus* Schönherr and *Ahasverus advena* were also collected at one of these sites.

##### Family Nitidulidae Latreille, 1802

The Nitidulidae of NB and the Maritime Provinces were first reviewed by Majka et al. (2008), where they newly recorded 28 species. Webster et al. (2012m) added another three species. Here, we add three more species, including *Stelidota coenosa* Erichson, which is newly recorded for Canada. Two of these species were captured almost exclusively in Lindgren funnel traps.

###### Subfamily Epuraeinae Kirejtshuk, 1986

####### Tribe Epuraeini Kirejtshuk, 1986

######## Epuraea linearis

Taxon classificationAnimaliaColeopteraNitidulidae

Mäklin, 1853*

######### Material examined.

**New Brunswick, Carleton Co.**, Jackson Falls, “Bell Forest Preserve”, 46.2210°N, 67.7210°W, 25.VII.2007, R.P. Webster // Rich Appalachian hardwood forest, m.v. light (1, RWC); Meduxnekeag Valley Nature Preserve, 46.1907°N, 67.6740°W, 17-31.VII.2012, C. Alderson & V. Webster // Old mixed forest, Lindgren funnel trap 1 m high under *Populus tremuloides* (1, AFC). **Queens Co.**, C.F.B. Gagetown, 45.7516°N, 66.1866°W, 9-22.V.2013, C. Alderson & V. Webster // Old mixed forest with *Quercus rubra*, Lindgren funnel trap in canopy of *Quercus rubra* (1, AFC). **Restigouche Co.**, Dionne Brook P.N.A., 47.9030°N, 68.3503°W, 9-23.VIII.2011, M. Roy & V. Webster // Old-growth northern hardwood forest, Lindgren funnel trap (1, RWC); same locality and collectors but 47.9064°N, 68.3441°W, 31.V-15.VI.2011, 28.VII-9.VIII,2011 // Old-growth white spruce & balsam fir forest, Lindgren funnel traps (2, RWC); Jacquet River Gorge P.N.A., 47.8257°N, 66.0764°W, 15-29.V.2014, 29.V-10.VI.2014, C. Alderson & V. Webster // Old *Populus balsamifera* stand near river, Lindgren funnel traps 1 m high under trees (1, AFC; 2, NBM). **Sunbury Co.**, Acadia Research Forest, 45.9866°N, 66.3841°W, 24-30.VI.2009, R. Webster & M.-A. Giguère // Red spruce forest with red maple & balsam fir, Lindgren funnel trap (1, RWC). **York Co.**, Charters Settlement, 45.8331°N, 66.7410°W, 27.VII.2005, R.P. Webster // Mixed forest, on flowers of *Spiraea alba* (1, RWC); Fredericton, Odell Park, 45.9571°N, 66.6650°W, 1-15.VI.2012, 28.VI-10.VII.2012, C. Alderson & V. Webster // Old-growth eastern hemlock forest, Lindgren funnel traps 1 m high under *Tsuga canadensis* (2) and *Betula alleghaniensis* (3) (1, AFC; 4, RWC); same locality and collectors but 45.9539°N, 66.6666°W, 15-27.V.2013, 27.V-10.VI.2013 // Hardwood stand, Lindgren funnel traps 1 m high under trees (2, AFC); Douglas, Currie Mountain, 45.9832°N, 66.7564°W, 24.VI-9.VII.2013, C. Alderson & V. Webster // Old *Pinus strobus* stand, Lindgren funnel trap in canopy of *Pinus strobus* (1, RWC); Canterbury, Eel River P.N.A., 45.8966°N, 67.6345°W, 8-21.V.2014, C. Alderson & V. Webster // Old-growth eastern white cedar swamp & fen, Lindgren funnel trap (1, NBM).

######### Distribution in Canada and Alaska.

AK, YT, NT, BC, AB, QC, **NB** (Bousquet et al. 2013).

######## Epuraea populi

Taxon classificationAnimaliaColeopteraNitidulidae

Dodge, 1939

######### Material examined.

**New Brunswick, Gloucester Co.**, Bathurst, Daly Point Nature Preserve, 47.6392°N, 65.6098°W, 13-28.V.2015, 25.VI-9.VII.2015, C. Alderson & V. Webster // Mixed forest, green (1) and black (1) Lindgren funnel traps in canopy (2, RWC). **Northumberland Co.**, ca. 1.5 km NW of Sevogle, 47.0939°N, 65.8387°W, 28.V-11.VI.2013, C. Alderson & V. Webster // *Populus tremuloides* stand with a few conifers, Lindgren funnel traps in canopy of *Populus tremuloides* (2, RWC). **Restigouche Co.**, Jacquet River Gorge P.N.A., 47.8257°N, 66.0764°W, 19.VIII-2.IX.2014, C. Alderson & V. Webster // Old *Populus balsamifera* stand near river, Lindgren funnel trap under trees (1, RWC). **York Co.**, Keswick Ridge, 45.9962°N, 66.8781°W, 22.V-4.VI.2014, C. Alderson & V. Webster // Field/meadow, Lindgren funnel trap 1 m high under trees (1, RWC).

######### Distribution in Canada and Alaska.

BC, AB, SK, MB, ON, QC, **NB** (Bousquet et al. 2013).

###### Subfamily Nitidulinae Latreille, 1802

####### Tribe Nitidulini Latreille, 1802

######## Stelidota coenosa

Taxon classificationAnimaliaColeopteraNitidulidae

Erichson, 1843

######### Material examined.

**Canada, New Brunswick, Northumberland Co.**, ca, 2.5 km W of Sevogle, 47.0876°N, 65.8613°W, 27.VIII.2013, R.P. Webster // Old *Pinus banksiana* forest, in partially dried boletus mushrooms (3, RWC).

######### Distribution in Canada and Alaska.

**NB (New Canadian record)**.

######### Comments.

Adults of *Stelidota coenosa* were collected from partially dried bolete mushrooms on a roadside through a jack pine forest. Little is known about the habitat requirements of this species. Other species in the genus have been found in decaying fruit and fungi (Downie and Arnett 1996). Most members of the genus are tropical (Ford 1996). In the United States, *Stelidota coenosa* (as *Stelidota ferruginea* Reitter) has been recorded from NJ west to MI, south to FL and TX (Downie and Arnett 1996).

##### Family Endomychidae Leach, 1815

Webster et al. (2012m) newly recorded *Hadromychys chandleri* Bousquet and Leschen and *Danae testacea* (Ziegler) for NB in their review of the NB members of the family. Here, we add two *Symbiotes* species to the faunal list. Both species were captured exclusively in Lindgren funnel traps.

###### Subfamily Anamorphinae Strohecker, 1953

####### Symbiotes duryi

Taxon classificationAnimaliaColeopteraEndomychidae

Blatchley, 1910

######## Material examined.

**New Brunswick, Carleton Co.**, Jackson Falls, “Bell Forest”, 46.2200°N, 67.7231°W, 19-28.VII.2008, R.P. Webster // Rich Appalachian hardwood forest, Lindgren funnel trap (1, RWC); same locality data, forest type and trap type but 16-21.VI.2009, R. Webster & M.-A. Giguère (1, RWC). **Gloucester Co.**, Bathurst, Daly Point Nature Preserve, 47.6392°N, 65.6098°W, 25.VI-9.VII.2015, 5-21.VIII.2015, C. Alderson & V. Webster // Mixed forest, purple Lindgren funnel traps in canopy of white pine (1), 1 m high (1) (2, AFC). **Kent Co.**, Kouchibouguac National Park, 46.8087°N, 64.9078°W, 27.V-12.VI.2015, 12-24.VI.2015, C. Alderson & V. Webster // Poplar/red maple stand, Lindgren funnel traps, 1 m high (2, AFC). **Northumberland Co.**, ca, 2.5 km W of Sevogle, 47.0876°N, 65.8613°W, 11-26.VI.2013, C. Alderson & V. Webster // Old *Pinus banksiana* stand, Lindgren funnel trap (1, AFC). **Queens Co.**, Cranberry Lake P.N.A., 46.1125°N, 65.6075°W, 12-21.V.2009, 21-27.V.2009, 11-18.VI.2009, 1-10.VII.2009, R.P. Webster & M.-A. Giguère // Old red oak forest, Lindgren funnel traps (7, RWC); same locality data, forest type and trapping method but 13-20.VII.2011, M. Roy & V. Webster (1, RWC); C.F.B. Gagetown, 45.7516°N, 66.1866°W, 22.V-4.VI.2013, C. Alderson & V. Webster // Old mixed forest with *Quercus rubra*, Lindgren funnel trap in canopy of *Quercus rubra* (1, AFC). **York Co.**, 15 km W of Tracy off Rt. 645, 45.6848°N, 66.8821°W, 11-19.V.2009, R.P. Webster & M.-A. Giguère // Old red pine forest, Lindgren funnel trap (1, RWC); Fredericton, Odell Park, 45.9539°N, 66.6666°W, 27.V-10.VI.2013, C. Alderson & V. Webster // Hardwood stand, Lindgren funnel trap in canopy (1, AFC).

######## Distribution in Canada and Alaska.

ON, **NB** (Bousquet et al. 2013).

####### Symbiotes gibberosus

Taxon classificationAnimaliaColeopteraEndomychidae

(Lucas, 1849)†

######## Material examined.

**New Brunswick, Queens Co.**, Cranberry Lake P.N.A., 46.1125°N, 65.6075°W, 28.VII-6.VIII.2009, R.P. Webster & M.-A. Giguère // Old red oak forest, Lindgren funnel traps (2, RWC); same locality data, forest type, and trapping method but 7-22.VI.2011, 22-29.VI.2011, 29.VI-7.VII.2011, 7-13.VII.2011, 13-20.VII.2011, M. Roy & V. Webster (8, RWC); C.F.B. Gagetown, 45.7516°N, 66.1866°W, 15-31.VII.2013, 17-30.VII.2015, C. Alderson & V. Webster // Old mixed forest with *Quercus rubra*, Lindgren funnel traps in canopy (1, AFC; 1, RWC). **York Co.**, Douglas, Currie Mountain, 45.9832°N, 66.7564°W, 27.V-10.VI.2013, C. Alderson & V. Webster // Old *Pinus strobus* stand, Lindgren funnel trap 1 m high under *Pinus strobus* (1, AFC).

######## Distribution in Canada and Alaska.

ON, **NB** (Bousquet et al. 2013).

######## Comments.

The Palaearctic *Symbiotes gibberosus* was first reported for Canada by Bousquet et al. (2013) without any supporting data. Klimaszewski et al. (2015) provided details for this record, which was from near St. Williams, ON. Specimens were collected in a forest near vernal pools. No information was provided on the habitat of this adventive species in the Palaearctic. Most specimens from NB were captured in an old red oak forest and one from an old white pine stand.

##### Family Coccinellidae Latreille, 1807

Majka and McCorquodale (2006) reported 39 species of Coccinellidae from NB but did not report any new provincial records. Recently, Webster et al. (2012m) reported three species new to the province. Here, we newly record *Diomus terminatus* (Say) and *Didion nanum* (LeConte).

###### Subfamily Coccinellinae Latreille, 1807

####### Tribe Diomini Gordon, 1999

######## Diomus terminatus

Taxon classificationAnimaliaColeopteraCoccinellidae

(Say, 1835)

######### Material examined.

**New Brunswick, Restigouche Co.**, Jacquet River Gorge P.N.A., 47.8200°N, 66.0015°W, 13.V.2010, R.P. Webster // Under alders in leaf litter & moss near small brook & *Carex* marsh (1, RWC).

######### Distribution in Canada and Alaska.

ON, QC, **NB** (Bousquet et al. 2013).

####### Tribe Scymnini Mulsant, 1846

######## Didion nanum

Taxon classificationAnimaliaColeopteraCoccinellidae

(LeConte, 1852)

######### Material examined.

**New Brunswick, Carleton Co.**, Jackson Falls, “Bell Forest”, 46.2200°N, 67.7231°W, 23.V-7.VI.2012, C. Alderson & V. Webster // Rich Appalachian hardwood forest, Lindgren funnel trap in canopy of *Tilia americana* (1, AFC); Meduxnekeag Valley Nature Preserve, 46.1907°N, 67.6740°W, 17-31.VII.2012, C. Alderson & V. Webster // Old mixed forest, Lindgren funnel trap in canopy of *Populus tremuloides* (1, AFC). **Gloucester Co.**, Bathurst, Daly Point Nature Preserve, 47.6392°N, 65.6098°W, 13-28.V.2015, 28.V-15.VI.2015, C. Alderson & V. Webster // Mixed forest, Lindgren funnel traps in canopy (2, AFC; 1, RWC). **Northumberland Co.**, ca. 1.5 km NW of Sevogle, 47.0939°N, 65.8387°W, 1-14.V.2013, C. Alderson & V. Webster // *Populus tremuloides* stand with a few conifers, Lindgren funnel trap 1 m high under *Populus tremuloides* (1, AFC). **Queens Co.**, Cranberry Lake P.N.A., 46.1125°N, 65.6075°W, 25.V-7.VI.2011, 7-22.VI.2011, 7-13.VII.2011, 13-20.VII.2011, 4-18.VIII.2011, 18-31.VIII.2011, M. Roy & V. Webster // Old red oak forest, Lindgren funnel traps in forest canopy (3, AFC; 7, RWC); C.F.B. Gagetown, 45.7516°N, 66.1866°W, 9-22.V.2013, C. Alderson & V. Webster // Old mixed forest with *Quercus rubra*, Lindgren funnel trap in canopy of *Quercus rubra* (1, AFC). **Restigouche Co.**, Jacquet River Gorge P.N.A., 47.8257°N, 66.0764°W, 29.V-10.VI.2014, 10-25.VI.2014, C. Alderson & V. Webster // Old *Populus balsamifera* stand near river, Lindgren funnel trap in canopy of *Populus balsamifera* (1, AFC; 1, RWC); ca. 3 km SE of Simpsons Field, 47.5277°N, 66.5142°W, 25.VI-10.VII.2015, C. Alderson & V. Webster // Old cedar & spruce forest with *Populus balsamifera* & *Populus tremuloides*, Lindgren funnel trap in canopy of *Populus balsamifera* (1, AFC). **Sunbury Co.**, Gilbert Island, 45.8770°N, 66.2954°W, 25.VII-8.VIII.2012, C. Alderson, C. Hughes, & V. Webster // Hardwood forest, Lindgren funnel traps in canopy of *Tilia amricana* (1, AFC). **York Co.**, 15 km W of Tracy off Rt. 645, 45.6848°N, 66.8821°W, 27.VII-10.VIII.2010, R.P. Webster & C. Hughes // Old red pine forest, Lindgren funnel trap (1, RWC); same locality and habitat data, and trapping method but 10-30.VIII.2010, C. Hughes & K. Burgess (1, AFC); Douglas, Currie Mountain, 45.9832°N, 66.7564°W, 3-15.V.2013, C. Alderson & V. Webster // Old *Pinus strobus* stand, Lindgren funnel trap 1 m high under *Pinus strobus* (1, AFC); Fredericton, Odell Park, 45.9539°N, 66.6666°W, 10-24.VI.2013, C. Alderson & V. Webster // Hardwood stand, Lindgren funnel trap in canopy (1, AFC); Keswick Ridge, 45.9962°N, 66.8781°W, 22.V-4.VI.2014, 13-29.VIII.2014, C. Alderson & V. Webster // Mixed forest, Lindgren funnel trap in canopy (1, RWC).

######### Distribution in Canada and Alaska.

ON, QC, **NB** (Bousquet et al. 2013).

######### Comments.

Most (22 out of 26) specimens of *Didion nanum* were captured in Lindgren funnel traps in the canopy of trees.

##### Family Corylophidae LeConte, 1852

The classification, taxonomy, and biology of the North American species of Corylophidae (minute hooded beetles or minute fungus beetles) were reviewed by Bowestead and Leschen (2002). Members of this family are often found on leaves and flowers, in leaf litter, grass piles, under bark, and sometimes in bird and caterpillar nests where the adults and larvae feed on fungal spores (Bowestead and Leschen 2002). Majka and Cline (2006) reviewed the Corylophidae of the Maritime Provinces and reported three species from NB, including *Orthoperus suturalis* LeConte and *Rypobius marinus* LeConte, which were new to the province. Bousquet et al. (2013) included another species on the faunal list of NB, *Clypastraea lugubris* (LeConte), in the most recent checklist of the Coleoptera of Canada. In this publication, we newly record five species of Corylophidae for NB.

###### Subfamily Corylophinae LeConte, 1852

####### Tribe Orthoperini Jacquelin du Val, 1857

######## Orthoperus scutellaris

Taxon classificationAnimaliaColeopteraCorylophidae

LeConte, 1878

######### Material examined.

**New Brunswick, Saint John Co.**, Chance Harbour, 45.1156°N, 66.3610°W, 7.V.2006, R.P. Webster // Sea beach, in decaying sea wrack on gravel and sand (1, RWC); Chance Harbour off Cranberry Head Rd., 45.1355°N, 66.3438°W, 12.VIII.2007, 12.V.2008, R.P. Webster // Barrier beach, in drift material and decaying sea wrack on gravel and sand (2, RWC). **Sunbury Co.**, Tracy, off Webb Rd., 45.6931°N, 66.6539°W, 31.VIII.2008, R.P. Webster // Mixed forest, sweeping roadside vegetation (1, RWC).

######### Distribution in Canada and Alaska.

NT, BC, AB, SK, ON, **NB**, NS (Bousquet et al. 2013).

######### Comments.

Most specimens of *Orthoperus scutellaris* were sifted from decaying sea wrack and drift material on sea beaches. One individual was swept from roadside vegetation.

####### Tribe Parmulini Poey, 1854

######## Clypastraea fusca

Taxon classificationAnimaliaColeopteraCorylophidae

(Harold, 1875)

######### Material examined.

**New Brunswick, Kent Co.**, Kouchibouguac National Park, 46.8072°N, 64.9100°W, 12-14.VI.2015, C. Alderson & V. Webster // Jackpine forest, Lindgren funnel trap, 1 m high (1, AFC). **Gloucester Co.**, Bathurst, Daly Point Nature Preserve, 47.6392°N, 65.6098°W, 13-28.V.2015, 15-25.VI.2015, C. Alderson & V. Webster // Mixed forest, purple Lindgren funnel trap in canopy (1, AFC). **Northumberland Co.**, ca, 2.5 km W of Sevogle, 47.0876°N, 65.8613°W, 28.V-11.VI.2013, 11-26.VI.2013, C. Alderson & V. Webster // Old *Pinus banksiana* stand, Lindgren funnel trap (1, AFC). **Queens Co.**, Cranberry Lake P.N.A., 46.1125°N, 65.6075°W, 11-25.V.2011, M. Roy & V. Webster // Old red oak forest, Lindgren funnel trap (1, RWC); C.F.B. Gagetown, 45.7516°N, 66.1866°W, 22.V-4.VI.2013, C. Alderson & V. Webster // Old mixed forest with *Quercus rubra*, Lindgren funnel trap in canopy of *Quercus rubra* (1, RWC). **Restigouche Co.**, Dionne Brook P.N.A., 47.9030°N, 68.3503°W, 30.V-15.VI.2011, M. Roy & V. Webster // Old-growth northern hardwood forest, Lindgren funnel trap (3 AFC: 1, NBM); same locality and collectors but 47.9064°N, 68.3441°W, 31.V-15.VI.2011, 15-27.VI.2011 // Old-growth white spruce & balsam fir forest, Lindgren funnel traps (4, RWC); ca. 3 km SE of Simpsons Field, 47.5277°N, 66.5142°W, 28.V-16.VI.2015, C. Alderson & V. Webster // Old cedar & spruce forest with *Populus balsamifera* & *Populus tremuloides*, Lindgren funnel traps (2, AFC). **Sunbury Co.**, Acadia Research Forest, 45.9866°N, 66.3441°W, 8-13.V.2009, 2-9.VI.2009, 13-21.VII.2009, R.P. Webster & M.-A. Giguère // Red spruce forest with red maple & balsam fir, Lindgren funnel traps (3, RWC); Gilbert Island, 45.8770°N, 66.2954°W, 18-28.V.2012, C. Alderson, C. Hughes, & V. Webster // hardwood forest, Lindgren funnel trap in canopy of *Tilia americana* (1, RWC). **York Co.**, 16 km W of Tracy off Rt. 645, 45.6855°N, 66.8847°W, 2-16.VI.2010, R.P. Webster & C. MacKay // Old red pine forest, Lindgren funnel trap (1, RWC)

######### Distribution in Canada and Alaska.

SK, ON, QC, **NB**, NS (Bousquet et al. 2013).

######### Comments.

All specimens of *Clypastraea fusca* were captured in Lindgren funnel traps in various forest types throughout the province.

######## Clypastraea lunata

Taxon classificationAnimaliaColeopteraCorylophidae

(LeConte, 1852)

######### Material examined.

**New Brunswick, Carleton Co.**, Jackson Falls, “Bell Forest”, 46.2200°N, 67.7231°W, 8-23.V.2012, C. Alderson & V. Webster // Rich Appalachian hardwood forest, Lindgren funnel trap in canopy of *Fagus grandifolia* (1, RWC). **Gloucester Co.**, Bathurst, Daly Point Nature Preserve, 47.6392°N, 65.6098°W, 13-28.V.2015, 15-25.VI.2015, C. Alderson & V. Webster // Mixed forest, black Lindgren funnel traps in canopy (2, AFC). **Kent Co.**, Kouchibouguac National Park, 46.8087°N, 64.9078°W, 21-27.V.2015, C. Alderson & V. Webster // Poplar/red maple stand, Lindgren funnel trap, 1 m high (1, AFC); same locality but 46.8072°N, 64.9100°W, 21-27.V.2015, C. Alderson & V. Webster // Jackpine forest, Lindgren funnel trap, 1 m high (1, AFC). **Northumberland Co.**, ca. 1.5 km NW of Sevogle, 47.0939°N, 65.8387°W, 1-14.V.2013, C. Alderson & V. Webster // *Populus tremuloides* stand with a few conifers, Lindgren funnel trap 1 m high under *Populus tremuloides* (1, AFC). **Queens Co.**, Cranberry Lake P.N.A., 46.1125°N, 65.6075°W, 5-12.V.2009, R.P. Webster & M.-A. Giguère // Old red oak forest, Lindgren funnel trap (1, RWC); same locality data, forest type, and collection method but 22-29.VI.2011, 20.VII-4.VIII.2011, 4-18.VIII.2011, M. Roy & V. Webster (4, RWC); Grand Lake Meadows P.N.A., 45.8227°N, 66.1209°W, 21.VI-5.VII.2011, M. Roy & V. Webster // Old silver maple forest with green ash and seasonally flooded marsh, Lindgren funnel trap (1, RWC); Jemseg, 45.8412°N, 66.1195°W, 14-28.V.2012, C. Alderson, C. Hughes, & V. Webster // Hardwood woodland near seasonally flooded marsh, Lindgren funnel trap 1 m high under *Rhus hirta* (1, AFC). **Restigouche Co.**, ca. 3 km SE of Simpsons Field, 47.5277°N, 66.5142°W, 14-28.V.2015, C. Alderson & V. Webster // Old cedar & spruce forest with *Populus balsamifera* & *Populus tremuloides*, Lindgren funnel trap (1, AFC). **Sunbury Co.**, Acadia Research Forest, 45.9866°N, 66.3441°W, 13-19.V.2009, R.P. Webster & M.-A. Giguère // Red spruce forest with red maple & balsam fir, Lindgren funnel trap (1, RWC); Sunpoke Lake, 45.7656°N, 66.5550°W, 3-15.VIII.2012, C. Alderson & V. Webster // Red oak forest near seasonally flooded marsh, Lindgren funnel trap in canopy of *Quercus rubra* (1, RWC). **York Co.**, Charters Settlement, 45.8395°N, 66.7391°W, 4.IV.2010, R.P. Webster // Mixed forest opening, collected with net during evening flight between 16:30 & 19:00h (2, RWC); Fredericton, Odell Park, 45.9539°N, 66.6666°W, 2-15.V.2013, C. Alderson & V. Webster // Hardwood stand, Lindgren funnel traps in canopy (1, AFC; 1, NBM); Douglas, Currie Mountain, 45.9832°N, 66.7564°W, 3-15.V.2013, C. Alderson & V. Webster // Old *Pinus strobus* stand, Lindgren funnel traps in canopy of *Pinus strobus* (1, AFC); Douglas, Currie Mountain, 45.9844°N, 66.7592°W, 3-15.V.2013, C. Alderson & V. Webster // Mixed forest with *Quercus rubra*, Lindgren funnel traps in canopy of *Quercus rubra* (1, AFC; 1, NBM).

######### Distribution in Canada and Alaska.

ON, **NB**, NS (Bousquet et al. 2013).

######### Comments.

All but one specimen of *Clypastraea lunata* were captured in Lindgren funnel traps. One individual was collected with an aerial net during an evening flight.

####### Tribe Peltinodini Paulian, 1950

######## Holopsis marginicollis

Taxon classificationAnimaliaColeopteraCorylophidae

(LeConte, 1852)

######### Material examined.

**New Brunswick, Carleton Co.**, Two Mile Brook Fen, 46.3619°N, 67.6733°W, 6.V.2005, M. Giguère & R. Webster // Forested cedar fen, in litter at base of cedar (4, RWC). **York Co.**, Charters Settlement, 45.8282°N, 66.7367°W, 16.IV.2005, R.P. Webster // *Carex* marsh, in sphagnum & litter at base of tree (3, RWC).

######### Distribution in Canada and Alaska.

MB, ON, QC, **NB** (Bousquet et al. 2013).

######### Comments.

Specimens of *Holopsis marginicollis* were sifted from litter and sphagnum at bases of trees in a *Carex* marsh and a forested cedar fen.

####### Tribe Sericoderini Matthews, 1888

######## Sericoderus lateralis

Taxon classificationAnimaliaColeopteraCorylophidae

(Gyllenhal, 1827)†

######### Material examined.

**New Brunswick, Queens Co.**, Cranberry Lake P.N.A., 46.1125°N, 65.6075°W, 4-18.VIII.2011, M. Roy & V. Webster // Old red oak forest, Lindgren funnel trap (1, RWC). **York Co.**, Charters Settlement, 45.8395°N, 66.7391°W, 16.IX.2005, 5.X.2007, 8.VIII.2010, 22.IX.2010, R.P. Webster // Mixed forest, in decaying (moldy) corncobs & cornhusks (7, RWC).

######### Distribution in Canada and Alaska.

BC, MB, ON, QC, **NB**, NS (Bousquet et al. 2013).

######### Comments.

Most specimens of this adventive species were found in a pile of decaying (moldy) corncobs and cornhusks. One individual was captured in a Lindgren funnel trap.

##### Family Latridiidae Erichson, 1842

Webster et al. (2012m) newly recorded nine species of Latridiidae for NB in their review of this family. Klimaszewski et al. (2015) noted that the specimen reported as *Stephostethus productus* Rosenhauer by Webster et al. (2012m) was misidentified. Details on this are provided below.

###### Subfamily Latridiinae Erichson, 1842

####### *Stephostethus productus* Rosenhauer, 1856

The specimen of *Stephostethus productus* reported from Tracy, NB as a new Canadian record by Webster et al. (2012m) was misidentified and was *Latridius hirtus* Gyllenhal. *Stephostethus productus* is accordingly removed from the faunal list of Canada and NB. However, *Latridius hirtus* is a new record for NB.

####### Latridius hirtus

Taxon classificationAnimaliaColeopteraLatridiidae

Gyllenhal, 1827†

######## Material examined.

**New Brunswick, Northumberland Co.**, ca. 1.5 km NW of Sevogle, 47.0939°N, 65.8387°W, 11-26.VI.2013, C. Alderson & V. Webster // *Populus tremuloides* stand with a few conifers, Lindgren funnel traps in canopy of *Populus tremuloides* (1, RWC). **York Co.**, 15 km W of Tracy off Rt. 645, 45.6848°N, 66.8821°W, 8-15.VI.2009, R. Webster & M.-A. Giguère // Old red pine forest, Lindgren funnel trap (1, RWC).

######## Distribution in Canada and Alaska.

BC, MB, ON, QC, **NB** (Bousquet et al. 2013).

#### Superfamily Tenebrionoidea Latreille, 1802

##### Family Tetratomidae Billberg, 1820

Seven species of Tetratomidae were recorded for the first time for NB by Webster et al. (2012n) in their review of the species of this family occurring in the province. Here, four species are added to the provincial faunal list and one is removed as a result of a misidentification.

###### Subfamily Tetratominae Billberg, 1820

####### Tetratoma (Abstrulia) variegata Casey, 1900

*Tetratoma variegata* Casey was newly recorded for NB by Webster et al. (2012n). Re-examination of these specimens and additional specimens from two new sites, in reference to the descriptions of *Tetratoma variegata* and *Tetratoma canadensis* Nikitsky and Chantal in Nikitsky (2004), revealed that the original determination by Webster was incorrect and that these were *Tetratoma canadensis*. *Tetratoma variegata* is therefore removed from the faunal list of NB. *Tetratoma canadensis* is a new provincial record and details are provided below.

####### Tetratoma (Abstrulia) canadensis

Taxon classificationAnimaliaColeopteraTetratomidae

Nikitsky & Chantal, 2004

######## Material examined.

**New Brunswick, Northumberland Co.**, ca, 2.5 km W of Sevogle, 47.0876°N, 65.8613°W, 28.V-11.VI.2013, 11-26.VI.2013, 13-27.V.2014, C. Alderson & V. Webster // Old *Pinus banksiana* stand, Lindgren funnel traps (4, AFC; 1, NBM; 3, RWC); Upper Graham Plains, 47.1001°N, 66.8154°W, 28.V-10.VI.2014, 9-24.VII.2014, C. Alderson & V. Webster // Old black spruce forest, Lindgren funnel traps (3, AFC; 2, NBM: 3, RWC). **Restigouche, Co.**, Dionne Brook P.N.A., 47.9064°N, 68.3441°W, 31.V-15.VI.2011, 27.VI-14.VII.2011, 28.VII-9.VIII.2011, M. Roy & V. Webster, old-growth white spruce and balsam fir forest, Lindgren funnel traps (4, AFC; 1, NBM; 5, RWC); ca. 3 km SE of Simpsons Field, 47.5277°N, 66.5142°W, 28.V-16.VI.2015, C. Alderson & V. Webster // Old cedar & spruce forest with *Populus balsamifera* & *Populus tremuloides*, Lindgren funnel trap (1, AFC).

######## Distribution in Canada and Alaska.

QC, **NB** (Bousquet et al. 2013).

###### Subfamily Hallomeninae Gistel, 1848

####### Hallomenus (Hallomenus) binotatus

Taxon classificationAnimaliaColeopteraTetratomidae

(Quensel, 1790)†

######## Material examined.

**New Brunswick, Queens Co.**, Jemseg, 45.8412°N, 66.1195°W, 8-21.VIII.2012, C. Alderson, C. Hughes, & V. Webster // Hardwood woodland near seasonally flooded marsh, Lindgren funnel trap 1 m high under *Quercus macrocarpa* (1, RWC). **York Co.**, Keswick Ridge, 45.9962°N, 66.8781°W, 27.VIII-9.IX.2015, C. Alderson & V. Webster // Mixed forest, Lindgren funnel trap 1 m high under trees (1, RWC).

######## Distribution in Canada and Alaska.

AK, BC, MB, ON, QC, **NB** (Bousquet et al. 2013).

####### Hallomenus (Hallomenus) debilis

Taxon classificationAnimaliaColeopteraTetratomidae

LeConte, 1866

######## Material examined.

**New Brunswick, York Co.**, Charters Settlement, 45.8395°N, 66.7391°W, 17.VII.2007, R.P. Webster, coll. // Mixed forest, m.v. light (1, RWC)

######## Distribution in Canada and Alaska.

MB, QC, **NB** (Bousquet et al. 2013).

####### Hallomenus (Hallomenus) scapularis

Taxon classificationAnimaliaColeopteraTetratomidae

Melsheimer, 1846

######## Material examined.

**New Brunswick, Carleton Co.**, Meduxnekeag Valley Nature Preserve, 46.1907°N, 67.6740°W, 8.VIII.2006, R.P. Webster // Mixed forest, in slightly decayed polypore mushroom on log (1, RWC). **Queens Co.**, Cranberry Lake P.N.A., 46.1125°N, 65.6075°W, 28.VII-6.VIII.2009, R.P. Webster & M.-A. Giguère // Old red oak forest, Lindgren funnel trap (1, RWC); same locality data and forest type, 7-22.VII.2011, M. Roy & V. Webster // Lindgren funnel trap (1, RWC); same locality data and forest type, 18.VIII.2011, R.P. Webster // in *Laetiporus sulphureus* (chicken mushroom) on dead standing red oak (4, RWC). **Sunbury Co.**, Acadia Research Forest, 45.9990°N, 66.2823°W, 7-22.VIII.2012, C. Alderson & V. Webster // Mature balsam fir forest with scattered red spruce & red maple, Lindgren funnel trap (1, RWC).

######## Distribution in Canada and Alaska.

BC, ON, QC, **NB**, NS (Bousquet et al. 2013).

##### Family Melandryidae Leach, 1815

Webster et al. (2012n) reviewed the Melandryidae of NB, adding 10 species to the provincial list. Here, we add three more species, all captured in Lindgren funnel traps.

###### Subfamily Melandryinae Leach, 1815

####### Tribe Hypulini Gistel, 1848

######## Microtonus sericans

Taxon classificationAnimaliaColeopteraMelandryidae

LeConte, 1862

######### Material examined.

**New Brunswick, Kent Co.**, Kouchibouguac National Park, 46.8072°N, 64.9100°W, 22.VII-4.VIII.2015, C. Alderson & V. Webster // Jackpine forest, Lindgren funnel trap, 1 m high (1, RWC). **Northumberland Co.**, ca, 2.5 km W of Sevogle, 47.0876°N, 65.8613°W, 26.VI-8.VII.2013, 25.VI-9.VII.2014, 9-23.VII.2014, 6-20.VIII.2014, C. Alderson & V. Webster // Old *Pinus banksiana* stand, Lindgren funnel traps (1, AFC; 1, NBM; 6, RWC); ca. 1.5 km NW of Sevogle, 47.0939°N, 65.8387°W, 11-26.VI.2013, 8-22.VII.2013, C. Alderson & V. Webster // *Populus tremuloides* stand with a few conifers, Lindgren funnel traps in canopy of *Populus tremuloides* (1, AFC; 1, RWC).

######### Distribution in Canada and Alaska.

NS, **NB** (Bousquet et al. 2013).

####### Tribe Melandryini Leach, 1815

######## Emmesa blackmani

Taxon classificationAnimaliaColeopteraMelandryidae

(Hatch, 1927)

######### Material examined.

**New Brunswick, Gloucester Co.**, Bathurst, Daly Point Nature Preserve, 47.6392°N, 65.6098°W, 9-23.VII.2015, 23.VII-5.VIII.2015, C. Alderson & V. Webster // Mixed forest, Lindgren funnel traps 1 m high (2, RWC). **York Co.**, Keswick Ridge, 45.9962°N, 66.8781°W, 3-18.VII.2014, 18-30.VII.2014, C. Alderson & V. Webster // Mixed forest, Lindgren funnel traps 1 m high under trees (1, AFC; 1, RWC).

######### Distribution in Canada and Alaska.

QC, **NB** (Bousquet et al. 2013).

####### Tribe Serropalpini Latreille, 1829

######## Phloiotrya concolor

Taxon classificationAnimaliaColeopteraMelandryidae

(LeConte, 1866)

######### Material examined.

**New Brunswick, Northumberland Co.**, ca. 1.5 km NW of Sevogle, 47.0939°N, 65.8387°W, 22.VII-6.VIII.2013, C. Alderson & V. Webster // *Populus tremuloides* stand with a few conifers, Lindgren funnel trap in canopy of *Populus tremuloides* (1, RWC). **Queens Co.**, Cranberry Lake P.N.A., 46.1125°N, 65.6075°W, 29.VI-7.VII.2011, 4-18.VIII.2011, M. Roy & V. Webster // Old red oak forest, Lindgren funnel traps (2, RWC); C.F.B. Gagetown, 45.7516°N, 66.1866°W, 4-17.VI.2013, 17.VI-3.VII.2013, 3-15.VII.2013, 15-31.VII.2013, C. Alderson & V. Webster // Old mixed forest with *Quercus rubra*, Lindgren funnel traps in canopy of *Quercus rubra* (3), in canopy of *Populus grandifolia* (4) (2, AFC; 5, RWC). **Sunbury Co.**, Gilbert Island, 45.8770°N, 66.2954°W, 11-25.VII.2012, 20.VI-5.VII.2013, C. Alderson, C. Hughes, & V. Webster // hardwood forest, Lindgren funnel traps in canopy of *Juglans cinerea* and canopy of *Fraxinus pennsylvanica* (2, RWC).

######### Distribution in Canada and Alaska.

QC, **NB** (Bousquet et al. 2013).

######### Comments.

Most (10 out of 12) individuals of this species were captured in Lindgren funnel traps in the canopy of trees.

##### Family Mordellidae Latreille, 1802

Webster et al. (2012o) newly reported 11 species of Mordellidae for NB, including *Falsomordellistens pubescens* (Fabricius), which was new to Canada. Here, we add two more species to the provincial list. All specimens of these two species were captured in Lindgren funnel traps.

###### Subfamily Mordellinae Latreille, 1802

####### Tribe Mordellistenini Ermisch, 1941

######## Mordellistena fulvicollis

Taxon classificationAnimaliaColeopteraMordellidae

(Melsheimer, 1845)

######### Material examined.

**New Brunswick, Queens Co.**, C.F.B. Gagetown, 45.7516°N, 66.1866°W, 17.VI-3.VII.2013, 3-15.VII.2013, 15-31.VII.2013, C. Alderson & V. Webster // Old mixed forest with *Quercus rubra*, Lindgren funnel trap in canopy of *Quercus rubra* (2, AFC; 1, NBM; 5, RWC). **Sunbury Co.**, Gilbert Island, 45.8770°N, 66.2954°W, 29.VI-11.VII.2012, 11-25.VII.2012, 25.VII-8.VIII.2012, C. Alderson, C. Hughes, & V. Webster // hardwood forest, Lindgren funnel traps in canopy of *Juglans cinerea* (2), and *Tilia americana* (4) (2, AFC; 1, NBM; 3, RWC). **York Co.**, Fredericton, Odell Park, 45.9539°N, 66.6666°W, 24.VII-7.VIII.2013, C. Alderson & V. Webster // Hardwood stand, Lindgren funnel trap in canopy (1, RWC).

######### Distribution in Canada and Alaska.

ON, **NB** (Bousquet et al. 2013).

######### Comments.

All specimens of this species were captured in Lindgren funnel traps in the canopy of trees.

######## Pseudotolida arida

Taxon classificationAnimaliaColeopteraMordellidae

(LeConte, 1862)

######### Material examined.

**New Brunswick, Carleton Co.**, Jackson Falls, “Bell Forest”, 46.2200°N, 67.7231°W, 17-31.VII.2012, 31.VII-13.VIII.2012, C. Alderson & V. Webster // Rich Appalachian hardwood forest, Lindgren funnel traps in canopy of *Fagus grandifolia* and *Acer saccaharum* (2, RWC). **Queens Co.**, Cranberry Lake P.N.A., 46.1125°N, 65.6075°W, 21-28.VII.2009, R.P. Webster & M.-A. Giguère // Old red oak forest, Lindgren funnel trap (1, RWC). **Sunbury Co.**, Sunpoke Lake, 45.7656°N, 66.5550°W, 9-20.VII.2012, C. Alderson & V. Webster // Red oak forest near seasonally flooded marsh, Lindgren funnel trap in canopy of *Quercus rubra* (1, RWC). Gilbert Island, 45.8770°N, 66.2954°W, 5-17.VII.2013, C. Alderson, C. Hughes, & V. Webster // hardwood forest, Lindgren funnel trap in canopy of *Populus tremuloides* (1, RWC). **York Co.**, Douglas, Currie Mountain, 45.9832°N, 66.7564°W, 9-24.VII.2013, 24.VII-7.VIII.2013, C. Alderson & V. Webster // Old *Pinus strobus* stand, Lindgren funnel trap in canopy of *Pinus strobus* (2, AFC, 1, RWC); same locality and collectors but 45.9844°N, 66.7592°W, 9-24.VII.2013, 24.VII-7.VIII.2013 // Mixed forest with *Quercus rubra*, Lindgren funnel trap in canopy of *Quercus rubra* (1) and 1 m high under *Quercus rubra* (1) (2, RWC); Fredericton, Odell Park, 45.9539°N, 66.6666°W, 7-19.VIII.2013, C. Alderson & V. Webster // Hardwood stand, Lindgren funnel trap in canopy (1, RWC).

######### Distribution in Canada and Alaska.

ON, QC, **NB** (Bousquet et al. 2013).

######### Comments.

All specimens of *Pseudotolida arida* were captured in Lindgren funnel traps, most (9 out of 11) in traps deployed in the canopy of trees.

##### Family Tenebrionidae Latreille, 1802

Thirteen species of Tenebrionidae were newly reported for NB by Webster et al. (2012q). *Platydema exacavtum* (Say) was removed from the list of Tenebrionidae known from NB, and the presence of *Platydema americanum* Laporte and Brullé was confirmed. Later, Bousquet and Bouchard (2014), in a review of the *Paratenetus* of North America, described *Platydema exutus* Bousquet and Bouchard from Tabusintac, NS (should be NB), and included many localities for this species from the province. Here, we add *Cynaeus angustus* (LeConte) and the adventive *Gnathocerus cornutus* (Fabricius) to the faunal list of the province.

###### Subfamily Diaperinae Latreille, 1802

####### Tribe Diaperini Latreille, 1802

######## Cynaeus angustus

Taxon classificationAnimaliaColeopteraTenebrionidae

(LeConte, 1851)

######### Material examined.

**New Brunswick, York Co.**, Keswick Ridge, 45.9962°N, 66.8781°W, 18-30.VII.2015, C. Alderson & V. Webster // Mixed forest, Lindgren funnel trap in canopy (1, RWC).

######### Distribution in Canada and Alaska.

BC, AB, SK, MB, ON, QC, **NB** (Bousquet et al. 2013).

######## Gnathocerus cornutus

Taxon classificationAnimaliaColeopteraTenebrionidae

(Fabricius, 1798)

######### Material examined.

**New Brunswick, York Co.**, Fredericton, 20.XII.2011, L. Leger // Warehouse, in flour (4, AFC; 6, RWC).

######### Distribution in Canada and Alaska.

BC, MB, ON, QC, **NB**, NS (Bousquet et al. 2013).

##### Family Mycteridae Oken, 1843

*Lacconotus punctatus* (LeConte) is the only known member of the family Mycteridae (the palm and flower beetles) known from the Maritime Provinces. Majka and Selig (2006) reported it for the first time for the region from NS. Later, Webster et al. (2012p) reported it from three localities in NB. Here, we present additional records of this rare species from the province.

###### Subfamily Eurypinae J. Thomson, 1860

####### Lacconotus punctatus

Taxon classificationAnimaliaColeopteraMycteridae

LeConte, 1862

######## Material examined.

**Additional New Brunswick records, Queens Co.**, Jemseg, 45.8412°N, 66.1195°W, 24.V-12.VI.2012, C. Alderson, C. Hughes, & V. Webster // Hardwood woodland near seasonally flooded marsh, Lindgren funnel trap 1 m high under *Quercus macrocarpa* (1, AFC); C.F.B. Gagetown, 45.7516°N, 66.1866°W, 22.V-4.VI.2013, C. Alderson & V. Webster // Old mixed forest with *Quercus rubra*, Lindgren funnel trap in canopy of *Quercus rubra* (1, AFC). **Restigouche Co.**, Jacquet River Gorge P.N.A., 47.8257°N, 66.0764°W, 10-25.VI.2014, C. Alderson & V. Webster // Old *Populus balsamifera* stand near river, Lindgren funnel trap in canopy of *Populus balsamifera* (1, RWC). **Sunbury Co.**, Gilbert Island, 45.8770°N, 66.2954°W, 18-28.V.2012, 28.V-12.VI.2012, C. Alderson, C. Hughes, & V. Webster // Hardwood forest, Lindgren funnel traps in canopy of *Juglans cinerea* (17), *Populus tremuloides* (1), and *Tilia americana* (6) (9, AFC; 4, CNC; 7, NBM; 4, RWC); Sunpoke Lake, 45.7656°N, 66.5550°W, 24.V-4.VI.2012, 4-18.VI.2012, C. Alderson & V. Webster // Red oak forest near seasonally flooded marsh, Lindgren funnel traps in canopy of *Quercus rubra* (6, AFC, 6, NBM, 4, RWC). **York Co.**, Fredericton, Odell Park, 45.9539°N, 66.6666°W, 9-24.VII.2013, C. Alderson & V. Webster // Hardwood stand, Lindgren funnel trap in canopy (1, AFC); Douglas, Currie Mountain, 45.9844°N, 66.7592°W, 24.VII-7.VIII.2013, C. Alderson & V. Webster // Mixed forest with *Quercus rubra*, Lindgren funnel trap in canopy of *Quercus rubra* (1, AFC).

######## Distribution in Canada and Alaska.

QC, NB, NS (Bousquet et al. 2013).

######## Comments.

Pollock and Majka (2012) reviewed the Nearctic *Lacconotus* and provided new data on distribution and habitat associations of *Lacconotus punctatus*. They showed that *Lacconotus punctatus* is more widespread in eastern North America than previously known. *Lacconotus punctatus* was reported from mixed forests, an oak savanna, and mature bottomland hardwood forests (Pollock and Majka 2012, Ulyshen et al. 2010). In NB, this species was found in similar forest types, including a hardwood bottomland woodland, a hardwood bottomland forest on an island, a *Populus balsamifera* stand near a river, mixed forests with red oak, a red oak forest, and an oak forest near a seasonally flooded marsh. Webster et al. (2012p) reported specimens from a bottomland (silver maple forest), a red oak forest, and a mature red spruce forest with scattered red maple. Most adults were collected early in the season from late May to mid-June, with a few captured as late as early August. *Lacconotus punctatus* was found at seven new localities in NB and appears to be widespread in the province.

All specimens from NB were captured in Lindgren funnel traps, showing the effectiveness of these traps for detecting this rare species. Interestingly, all but four of the 48 adults of *Lacconotus punctatus* were captured in the canopy of trees. In a mature bottomland hardwood forest in GA, five of six specimens were captured in flight intercept traps 15 m above the forest floor early in the season (Ulyshen et al. (2010). They suggested that *Lacconotus punctatus* might be a canopy and early seasonal specialist and the reason why this species is infrequently collected. Our data support their conclusion.

##### Family Pyrochroidae Latreille, 1806

Webster et al. (2012p) newly recorded three species of Pyrochroidae [*Neopyrochroa femoralis* (LeConte), *Pedilus canaliculatus* (LeConte), *Promorensia elegans* (Hentz)] for NB. Here, we add *Dendroides testaceus* LeConte to the faunal list of the province.

###### Subfamily Pyrochroinae, Latreille, 1806

####### Dendroides testaceus

Taxon classificationAnimaliaColeopteraPyrochroidae

LeConte, 1855

######## Material examined.

**New Brunswick, Restigouche Co.**, ca. 3 km SE of Simpsons Field, 47.5277°N, 66.5142°W, 10-23.VII.2015, 2-21.VIII.2015, C. Alderson & V. Webster // Old cedar & spruce forest with *Populus balsamifera* & *Populus tremuloides*, Lindgren funnel traps (2, RWC). **York Co.**, Canterbury, Eel River P.N.A., 45.8966°N, 67.6345°W, 15-28.VII.2014, C. Alderson & V. Webster // Old-growth eastern white cedar swamp & fen, Lindgren funnel trap (1 ♂ [dissected], RWC).

######## Distribution in Canada and Alaska.

AB, SK, MB, ON, QC, **NB** (Bousquet et al. 2013).

######## Comments.

*Dendroides testaceus* is readily separated from the similar *Dendroides concolor* by possessing piceous pigmentation areas on the wings; in *Dendroides concolor* the pigmentation areas of wings are testaceous (Young 1975).

##### Family Aderidae Csiki, 1909

The Aderidae occurring in NB were reviewed by Webster et al. (2012p). They newly reported three species for the province. Here, we report the adventive *Aderus populneus* (Creutzer) and the native *Vanonus calvescens* Casey for the first time for NB. All specimens were captured in Lindgren funnel traps.

###### Tribe Aderini Csiki, 1909

####### Aderus populneus

Taxon classificationAnimaliaColeopteraAderidae

(Creutzer, 1796)†

######## Material examined.

**New Brunswick, Queens Co.**, Jemseg, 45.8412°N, 66.1195°W, 2-14.V.2012, C. Hughes, & R.P. Webster // Hardwood woodland near seasonally flooded marsh, Lindgren funnel trap 1 m high under *Quercus rubra* (1, RWC).

######## Distribution in Canada and Alaska.

BC, MB, QC, **NB**, NS (Bousquet et al. 2013).

####### Vanonus calvescens

Taxon classificationAnimaliaColeopteraAderidae

Casey, 1895

######## Material examined.

**New Brunswick, Gloucester Co.**, Bathurst, Daly Point Nature Preserve, 47.6392°N, 65.6098°W, 23.VII-5.VIII.2015, C. Alderson & V. Webster // Mixed forest, black Lindgren funnel trap 1 m high (1, RWC). **Queens Co.**, Cranberry Lake P.N.A, 46.1125°N, 65.6075°W, 4-18.VIII.2011, M. Roy & V. Webster // mature red oak forest, Lindgren funnel trap (1, RWC); Jemseg, 45.8412°N, 66.1195°W, 28.VI-10.VII.2012, C. Alderson, C. Hughes, & V. Webster // Hardwood woodland near seasonally flooded marsh, Lindgren funnel trap 1 m high under *Quercus macrocarpa* (1, RWC). **Sunbury Co.**, Gilbert Island, 45.8770°N, 66.2954°W, 12-29.VI.2012, 11-25.VII.2012, 25.VII-8.VIII.2012, C. Alderson, C. Hughes, & V. Webster // Hardwood forest, Lindgren funnel traps in canopy of *Tilia americana* (8) and 1 m high under *Tilia americana* (2) (2, AFC; 1, CNC; 7, RWC). **York Co.**, Fredericton, Odell Park, 45.9571°N, 66.6650°W, 28.VI-10.VII.2012, C. Alderson & V. Webster // Old-growth eastern hemlock forest, Lindgren funnel trap 1 m high under *Betula alleghaniensis* (1, RWC).

######## Distribution in Canada and Alaska.

QC, **NB** (Bousquet et al. 2013).

##### Family Scraptiidae Gistel, 1848

The Scraptiidae (False flower beetles or Scraptiid beetles) is a small family of beetles widely distributed in North America. Pollock (2002) provided an overview of the taxonomy, classification, and known biology of this family. Adults of many species occur on flowers, often in abundance, and the larvae live under bark of trees (Young 1991, Majka and Pollock 2006). Little else is known about the biology. Twenty species are known from Canada (Bousquet et al. 2013). Majka and Pollock (2006) reported three species of Scraptiidae previously known from NB in their review of this and related families of the Maritime Provinces. Here, we newly record *Anaspis nigrina* Csiki for the province.

###### Subfamily Anaspidinae Mulsant, 1856

####### Tribe Anaspidini Mulsant, 1856

######## Anaspis nigrina

Taxon classificationAnimaliaColeopteraScraptiidae

Csiki, 1915

######### Material examined.

**New Brunswick, Kent Co.**, Kouchibouguac National Park, 46.8087°N, 64.9078°W, 12-24.VI.2015, C. Alderson & V. Webster // Poplar/red maple stand, Lindgren funnel trap, 1 m high (1, RWC); same locality but 46.8072°N, 64.9100°W, 27.V-12.VI.2015, C. Alderson & V. Webster // Jackpine forest, Lindgren funnel trap, 1 m high (1, AFC). **Northumberland Co.**, ca, 2.5 km W of Sevogle, 47.0876°N, 65.8613°W, 11-26.VI.2013, C. Alderson & V. Webster // Old *Pinus banksiana* stand, Lindgren funnel trap (1, AFC; 4, RWC); Upper Graham Plains, 47.1001°N, 66.8154°W, 10-24.VI.2014, C. Alderson & V. Webster // Old black spruce forest, Lindgren funnel trap (1, AFC). **York Co.**, Charters Settlement, 45.8430°N, 66.7275°W, 4.VII.2004, R.P. Webster // Regenerating mixed forest, sweeping (1, RWC); 15 km W of Tracy off Rt. 645, 45.6848°N, 66.8821°W, 19-25.V.2009, 25.V-1.VI.2009, R. Webster & M.-A. Giguère // Old red pine forest, Lindgren funnel traps (2, RWC); 16 km W of Tracy off Rt. 645, 45.6855°N, 66.8847°W, 2-16.VI.2010, R. Webster & C. MacKay // Old red pine forest, Lindgren funnel trap (2, RWC); Fredericton, Odell Park, 45.9539°N, 66.6666°W, 9-24.VII.2013, C. Alderson & V. Webster // Hardwood stand, Lindgren funnel trap in canopy (1, RWC).

######### Distribution in Canada and Alaska.

BC, ON, QC, **NB**, NS, NF (Bousquet et al. 2013).

#### Superfamily Chrysomeloidea Latreille, 1802

The Megalopodidae and Chrysomelidae occurring in NB were reviewed by Webster et al. (2012b). *Zeugophora varians* Crotch was newly recorded for NB and represented the first record of the Megalopodidae for the province. They also newly recorded 28 species of Chrysomelidae for the province. Here, we add another three species of Megalopodidae, all captured in Lindgren funnel traps in the canopy of trees, and nine species of Chrysomelidae.

##### Family Megalopodidae Latreille, 1802

###### Subfamily Zeugophorinae Böving & Craighead, 1931

####### Zeugophora abnormis

Taxon classificationAnimaliaColeopteraMegalopodidae

(LeConte, 1850)

######## Material examined.

**New Brunswick, Carleton Co.**, Jackson Falls, “Bell Forest”, 46.2200°N, 67.7231°W, 21.VI-3.VII.2012, C. Alderson & V. Webster // Rich Appalachian hardwood forest, Lindgren funnel trap in canopy of *Juglans cinerea* (1, RWC). **Gloucester Co.**, Bathurst, Daly Point Nature Preserve, 47.6392°N, 65.6098°W, 13-28.V.2015, 18.V-15.VI.2015, C. Alderson & V. Webster // Mixed forest, green Lindgren funnel traps in canopy (5), black Lindgren funnel traps in canopy (3) (5, AFC; 3 RWC). **Queens Co.**, C.F.B. Gagetown, 45.7516°N, 66.1866°W, 4-17.VI.2013, C. Alderson & V. Webster // Old mixed forest with *Quercus rubra*, Lindgren funnel trap in canopy of *Populus tremuloides* (1, RWC). **Restigouche Co.**, Jacquet River Gorge P.N.A., 47.8257°N, 66.0764°W, 10-25.VI.2014, C. Alderson & V. Webster // Old *Populus balsamifera* stand near river, Lindgren funnel trap in canopy of *Populus balsamifera* (1, AFC). **Sunbury Co.**, Gilbert Island, 45.8770°N, 66.2954°W, 23.V-6.VI.2013, C. Alderson, C. Hughes, & V. Webster // hardwood forest, Lindgren funnel trap in canopy *Populus tremuloides* (1, RWC). **York Co.**, Keswick Ridge, 45.9962°N, 66.8781°W, 22.V-4.VI.2014, 4-19.VI.2014, 19.VI-3.VII.2014, 28.VIII-11.IX.2014, C. Alderson & V. Webster // Mixed forest, Lindgren funnel traps in canopy of *Populus tremuloides* (2, AFC; 4, RWC).

######## Distribution in Canada and Alaska.

AK, YT, NT, BC, AB, SK, MB, ON, QC, **NB**, NS, PE (Bousquet et al. 2013).

####### Zeugophora puberula

Taxon classificationAnimaliaColeopteraMegalopodidae

Crotch, 1873

######## Material examined.

**New Brunswick, Gloucester Co.**, Bathurst, Daly Point Nature Preserve, 47.6392°N, 65.6098°W, 15-25.VI.2015, C. Alderson & V. Webster // Mixed forest, green Lindgren funnel trap in canopy (1, AFC). **York Co.**, Douglas, Currie Mountain, 45.9832°N, 66.7564°W, 3-15.X.2013, C. Alderson & V. Webster // Old *Pinus strobus* stand, Lindgren funnel trap in canopy of *Pinus strobus* (1, RWC); Keswick Ridge, 45.9962°N, 66.8781°W, 18-30.VII.2014, C. Alderson & V. Webster // Mixed forest, Lindgren funnel trap in canopy (1, RWC); Douglas, N.B. Walking Trail, 45.9819°N, 66.7568°W, 20.IV-5.V.2015, C. Alderson & V. Webster // Hardwood forest, Lindgren funnel trap in canopy (2, RWC).

######## Distribution in Canada and Alaska.

MB, ON, QC, **NB** (Bousquet et al. 2013).

####### Zeugophora scutellaris

Taxon classificationAnimaliaColeopteraMegalopodidae

Suffrian, 1840†

######## Material examined.

**New Brunswick, Sunbury Co.**, Gilbert Island, 45.8770°N, 66.2954°W, 11-25.VII.2012, C. Alderson, C. Hughes, & V. Webster // hardwood forest, Lindgren funnel trap in canopy of *Populus tremuloides* (1, RWC). **York Co.**, Douglas, Currie Mountain, 45.9832°N, 66.7564°W, 24.VI-9.VII.2013, C. Alderson & V. Webster // Old *Pinus strobus* stand, Lindgren funnel traps in canopy of *Pinus strobus* (1, RWC).

######## Distribution in Canada and Alaska.

NT, BC, AB, SK, MB, ON, QC, **NB**, NS (Bousquet et al. 2013).

##### Family Chrysomelidae Latreille, 1802

###### Subfamily Bruchiniae Latreille, 1802

####### Tribe Bruchini Latreille, 1802

######## Subtribe Acanthoscelidina Bridwell, 1946

######### Bruchidius villosus

Taxon classificationAnimaliaColeopteraChrysomelidae

(Fabricius, 1775)†

########## Material examined.

**New Brunswick, Albert Co.**, Waterside Beach, 45.6282°N, 64.8129°W, 29.VI.2014, R.P. Webster // Sand dune, sweeping dune vegetation with beach pea (7, NBM; 8, RWC). **Sunbury Co.**, Maugerville, off Rt. 105, 45.8662°N, 66.4559°W, 4.VI.2014, 9.VI.2014, R.P. Webster // Flood plain forest, sweeping roadside foliage (1, NBM; 1, RWC). **York Co.**, Lincoln, 45.9120°N, 66.6115°W, 7.VI.2015, R.P. Webster // Meadow with clover & alfalfa, sweeping (1, RWC); Keswick Ridge, 45.9962°N, 66.8781°W, 2-18.VI.2015, C. Alderson & V. Webster // Mixed forest, Lindgren funnel trap in canopy (1, AFC).

########## Distribution in Canada and Alaska.

ON, QC, **NB**, NS (Bousquet et al. 2013).

########## Comments.

The adventive *Bruchidius villosus* (broom seed beetle) was first reported in Atlantic Canada from NS by Majka and Langor (2011). In NS, this adventive Palaearctic species was found on the adventive weed, Scotch broom (*Cytisis scoparius* (L.) Link) (Fabaceae) (Majka and Langor 2011). In NB, *Bruchidius villosus* was swept from foliage and flowers of *Lathyrus japonicus* Willid. (beach pea), a native member of the Fabaceae, and from foliage in a meadow, and along a roadside at two inland sites where beach pea and Scotch broom do not occur. More study is required to establish if *Bruchidius villosus* will become a pest on native Fabaceae.

###### Subfamily Galerucinae Latreille, 1802

####### Tribe Alticini Newman, 1834

######## Longitarsus ganglbauri

Taxon classificationAnimaliaColeopteraChrysomelidae

Heikertinger, 1873†

######### Material examined.

**New Brunswick, Saint John Co.**, Chance Harbour, off Cranberry Head Rd., 45.1355°N, 66.3438°W, 30.V.2006, R.P. Webster // Barrier beach, sweeping foliage of *Leucanthemum vulgare* Lam. (8, RWC).

######### Distribution in Canada and Alaska.

MB, **NB**, NS, PE (Bousquet et al. 2013).

######## Longitarsus rubiginosus

Taxon classificationAnimaliaColeopteraChrysomelidae

(Foudras, 1859)†

######### Material examined.

**New Brunswick, Charlotte Co.**, St. Andrews, 45.0741°N, 67.0383°W, 22.VII.2012, R.P. Webster // Barrier beach (gravel), under large log (4, RWC). **Queens Co.**, W of Jemseg at “Trout Creek”, 45.8237°N, 66.1225°W, R.P. Webster // Silver maple swamp, sweeping foliage along river margin (1, RWC).

######### Distribution in Canada and Alaska.

ON, QC, **NB**, NS (Bousquet et al. 2013).

######## Psylliodes picinus

Taxon classificationAnimaliaColeopteraChrysomelidae

(Marsham, 1802)†

######### Material examined.

**New Brunswick, Queens Co.**, Jemseg, 45.8412°N, 66.1195°W, 10-25.VII.2012, 25.VII-8.VIII.2012, C. Alderson, C. Hughes, & V. Webster // Hardwood woodland near seasonally flooded marsh, Lindgren funnel trap 1 m in *Rhus hirta* (1, RWC). **Sunbury Co.**, Gilbert Island, 45.8770°N, 66.2954°W, 11-25.VII.2012, 25.VII-8.VIII.2012, C. Alderson, C. Hughes, & V. Webster // hardwood forest, Lindgren funnel traps 1 m high under *Juglans cinerea* (1) *Tilia americana* (2) (3, RWC).

######### Distribution in Canada and Alaska.

ON, QC, **NB**, NS (Bousquet et al. 2013).

###### Subfamily Cryptocephalinae Gyllenhal, 1813

####### Tribe Cryptocephalini Gyllenhal, 1813

######## Pachybrachis (Pachybrachis) tridens

Taxon classificationAnimaliaColeopteraChrysomelidae

(Melsheimer, 1847)

######### Material examined.

**New Brunswick, York Co.** Keswick Ridge, 45.9962°N, 66.8781°W, 16-29.VII.2015, C. Alderson & V. Webster // Mixed forest, Lindgren funnel trap in canopy (1, RWC).

######### Distribution in Canada and Alaska.

MB, ON, QC, **NB** (Bousquet et al. 2013).

######### Comments.

Poison ivy, *Toxicodendron radicans* (L.) Kuntze, has been reported as the preferred host for this species (see Clark et al. (2004) for a complete list of references). This vine was common at the site where the NB specimen was collected. *Pachybrachis tridens* was thought to be restricted to the Carolinian Life Zone in Canada, and records from MB and QC were considered questionable by Barney et al. (2013). This distinctive species has not been collected in Canada since 1950 and was considered to be extirpated from Canada by Barney et al. (2013). The record from NB indicates that the species is still present in Canada.

####### Tribe Fulcidacini Jakobson, 1924

######## Neochlamisus bebbiane

Taxon classificationAnimaliaColeopteraChrysomelidae

(W.J. Brown, 1943)

######### Material examined.

**New Brunswick, Queens Co.**, Jemseg, 45.8412°N, 66.1195°W, 25.VII.2012, R.P. Webster // Hardwood woodland near seasonally flooded marsh, small pasture, sweeping foliage (1 ♀, RWC); Canning, Scotchtown near Indian Point, 45.8762°N, 66.1816°W, 5.VI.2004, R.P. Webster // Lake margin, oak/maple forest on sandy soil, sweeping foliage (1 ♀, NBM). **Sunbury Co.**, off Coy Rd., Grand Lake Meadows P.N.A., 45.9804°N, 66.1824°W, 20.VI.2013, R.P. Webster // Trail through mixed forest, sweeping vegetation; alders, willow, sweet-fern, blueberry (1 ♀, NBM). **York Co.**, New Maryland, 45.8390°N, 66.7389°W, 27.V.2003, R.P. Webster // Mixed forest, on foliage of alder (1 ♂, 1 ♀, RWC); Charters Settlement, 45.8430°N, 66.7275°W, 17.VI.2004, 27.VI.2004, 19.V.2005, 12.VII.2005, R.P. Webster // regenerating mixed forest, sweeping vegetation (5 ♂, 2 ♀, RWC); Keswick Ridge, 45.9962°N, 66.8781°W, 22.V-4.VI.2004, 4-19.VI.2014, C. Alderson & V. Webster // Mixed forest, Lindgren funnel trap 1 m high under trees (1, AFC).

######### Distribution in Canada and Alaska.

MB, ON, QC, **NB**, NS (Bousquet et al. 2013).

######## Neochlamisus chamaedaphnes

Taxon classificationAnimaliaColeopteraChrysomelidae

(W.J. Brown, 1943)

######### Material examined.

**New Brunswick, Sunbury Co.**, Bull Pasture Bog, 46.0354°N, 66.3358°W, 21.VI.2013, R.P. Webster // Moss lawn bog with black spruce & tamarack on margin, sweeping vegetation on bog margin (1 ♂, RWC).

######### Distribution in Canada and Alaska.

ON, QC, **NB**, NS (Bousquet et al. 2013).

###### Subfamily Eumolpinae Hope, 1840

####### Tribe Bromiini Baly, 1865

######## Xanthonia villosula

Taxon classificationAnimaliaColeopteraChrysomelidae

(Melsheimer, 1847)

######### Material examined.

**New Brunswick, Queens Co.**, C.F.B. Gagetown, 45.7516°N, 66.1866°W, 3-15.VII.2013, 17-30.VII.2015, C. Alderson & V. Webster // Old mixed forest with *Quercus rubra*, Lindgren funnel traps in canopy of *Quercus rubra* [1 male dissected] (5, RWC).

######### Distribution in Canada and Alaska.

ON, QC, **NB**, NS (Bousquet et al. 2013).

####### Tribe Typophorini Baly, 1865

######## Paria pratensis

Taxon classificationAnimaliaColeopteraChrysomelidae

Balsbaugh, 1970

######### Material examined.

**New Brunswick, Queens Co.**, Scotchtown near Indian Point [Grand Lake Meadows P.N.A.], 45.8762°N, 66.1816°W, 5.VI.2004, 16.VI.2013, R.P. Webster // Lake margin, oak maple forest on sandy soil, sweeping foliage (7, RWC). **Sunbury Co.**, Gilbert Island, 45.8770°N, 66.2954°W, 6.VI.2013, R.P. Webster // hardwood forest on island in river, sweeping vegetation (1, RWC); Grand Lake Meadows P.N.A., off Coy Rd., 45.9804°N, 66.1824°W, 20.VI.2013, R.P. Webster // Trail through mixed forest, sweeping vegetation (1, RWC). **Westmorland Co.**, Petit Cap, 46.1879°N, 64.1503°W, 17.VI.2014, M.-A. Giguère (1, NBM). **York Co.**, Charters Settlement, 45.8405°N, 66.7321°W, 8.VI.2003, R.P. Webster // Mixed forest, on foliage (1, RWC).

######### Distribution in Canada and Alaska.

ON, QC, **NB** (Bousquet et al. 2013).
